# Interventions delivered by primary or community healthcare professionals to support people living at home with dementia with activities of daily living: a systematic review and narrative synthesis

**DOI:** 10.1186/s12877-024-05465-5

**Published:** 2024-10-23

**Authors:** Helen Chester, Barbara Bradbury, Miriam Santer, Leanne Morrison, Mandy Fader, Jane Ward, Jill Manthorpe, Catherine Murphy

**Affiliations:** 1 NIHR Policy Research Unit in Health and Social Care Workforce, The Policy Institute King’s College London, Virginia Woolf Building 22 Kingsway, London, WC2B 6LE UK; 2 School of Health Sciences, University of Southampton, Southampton General Hospital, Tremona Road, Shirley, SO16 6YD UK; 3 Primary Care Research Centre, School of Primary Care, Population Sciences and Medical Education (PPM), Faculty of Medicine, University of Southampton, Aldermoor Health Centre, Southampton, SO16 5ST UK; 4 Department of Psychology, Faculty of Environmental and Life Sciences, Primary Care Research Centre, Faculty of Medicine, University of Southampton, Highfield Campus, Southampton, SO17 1BJ UK; 5 School of Health Sciences, University of Southampton University, Southampton General Hospital, Tremona Road, Southampton, SO16 6YD UK; 6 Lead for Dementia Friendly Hampshire/Alzheimer’s Society Research Network Member, Dementia Friendly Hampshire, Hampshire, UK

**Keywords:** Systematic review, People living with dementia, Family carers, Non-pharmacological interventions, Behavioural interventions, Continence, Healthcare professionals, Primary care, Community care

## Abstract

**Background:**

Most people living with dementia live in their own home supported by family carers. One of the most challenging problems they face is managing toilet-use and continence. Carers have repeatedly asked for better advice from healthcare professionals. The purpose of this systematic review was to inform the development of an intervention to support healthcare professionals to provide existing continence management advice to the carers of people living at home with dementia. It aimed to identify and synthesise lessons from the development and evaluation of interventions, involving primary or community healthcare professionals, to support the provision of management advice aimed at supporting people living at home with dementia and their carers with activities of daily living. Due to a lack of relevant continence or toilet-use interventions, this included, but was not limited to, toileting or continence care.

**Methods:**

Literature (February 2009-November 2022) was searched using five databases: MEDLINE (Ovid); PsycINFO (Ovid); EMBASE (Ovid); Cumulative Index to Nursing and Allied Health Literature (CINAHL) (EBSCO); and Cochrane Central Register of Controlled Trials (CENTRAL). Empirical studies using a variety of methodologies were included and thus the quality of papers appraised using the Mixed-Methods Appraisal Tool. No studies were excluded based on quality. A narrative synthesis was undertaken.

**Results:**

Twelve articles reporting on 10 interventions were included. Most comprised the provision of online resources only, although some combined these with online or face-to-face contact with healthcare professionals. A variety of methodologies was utilised including randomised controlled trials. The quality of included studies was variable. Six main themes were identified: mode of delivery; targeted and tailored resources; content, design and navigation; credibility; user involvement in the development and evaluation of information resources; and role of professionals and organisations.

**Conclusions:**

Despite the urgent need to better support people living at home with dementia and their carers, this review highlights the paucity of studies reporting on interventions delivered within primary and community healthcare contexts to provide management advice aimed at supporting this population with activities of daily living. This review has identified important considerations that will potentially aid the development, delivery and evaluation of such interventions.

**Systematic Review Registration:**

PROSPERO International Prospective Register of Systematic Reviews CRD42022372456.

**Supplementary Information:**

The online version contains supplementary material available at 10.1186/s12877-024-05465-5.

## Background

Worldwide dementia is a major cause of disability in older people (aged 65 and over) with more than 55 million people having dementia worldwide [1]. In the United Kingdom (UK) an estimated 61% of older people living with dementia live in their own homes [2]. Most people would rather live in their own home if diagnosed with dementia [3]. The majority of people living with dementia are supported by informal carers or caregivers, namely family members and friends [1] with approximately 700,000 carers supporting people living with dementia in the UK [4]. They provide a range of support with activities of daily living (ADLs) including personal care, and completing practical tasks such as shopping, meal preparation and laundry and instrumental activities of daily living (IADLs) such as medication management [5–7]. Carers should have access to evidence-based information and support so that they can provide good care and manage the demands of their caring role to enable people living with dementia to live well at home for as long as possible [8]. This also includes information about how to manage behaviour which impacts on the performance of basic and instrumental ADLs [9].

One of the most common problems for people living with dementia, and their carers, is managing toilet-use and continence problems and these problems can have a substantial negative impact on either or both parties [10, 11]. It can give rise to health problems, such as infections or skin breakdown, psychological problems including anxiety and depression, and can also increase stigma, social isolation and breakdown in care at home [10, 12]. Retaining the ability to independently use the toilet is the ADL rated most important by carers [13]. Despite this evidence, carers have repeatedly called for improved support to help them cope with continence needs [10, 11]. Specifically, they have stated that they would benefit from proactive input (including information) from the healthcare professionals with whom they come into contact [10, 14]. Many have asked for help in managing specific continence needs (e.g. containing bladder and bowel leakage), but also for continence related concerns linked to behavioural challenges, such as apathy or repetitive toilet-use. In response to this need, following a qualitative study with carers, people living with dementia, and nurses [10, 15], and reviewing available evidence, we developed detailed practical guidance to support carers with continence care. However, it was also clear from this work that many carers wanted healthcare professionals to be proactive in talking about continence problems, but healthcare professionals did not feel equipped to do so [15]. Therefore, we planned the development of an intervention to improve the continence care support provided by healthcare professionals for people living with dementia and their carers. Recognising the time limitations facing healthcare professionals, we accepted that an implementable intervention would need to be low-intensity, involving little direct communication. We also recognised that the evidence to support healthcare interventions for ADLs is sparse and inconclusive [16, 17]. We further acknowledged that many interventions developed to support people living at home with dementia and their carers can be ineffective if they fail to consider the complexities of living with the condition and are not tailored to the person with dementia and their carer’s needs [18, 19].

Given the challenges of developing an effective and acceptable intervention, the review reported in this paper was undertaken to identify and synthesise lessons from previous studies.

## Methods

A systematic review was conducted to identify and synthesise lessons from the development and evaluation of interventions, involving primary or community healthcare professionals, to support the provision of management advice aimed at supporting people living at home with dementia and their carers with activities of daily living. It took place as part of a wider programme of research conducted to develop a new intervention to support healthcare professionals to provide continence management advice to carers of people living at home with dementia [14]. Earlier research informed the written content for the website [15] which was refined through qualitative interviews and discussions with public contributors and stakeholders that also took place as part of this research programme [14, 20]. This systematic review took place alongside these elements. Because of a dearth of other studies to learn from regarding continence interventions, we took a broader view to learn from studies where carers were being helped to support people living with dementia with daily activities or challenges, including but not limited to, toileting or continence care. We were able to do this because the continence specific advice had already been developed as part of our wider research programme and the focus of this study was on the delivery of that advice. Therefore, the review primarily sought to identify and synthesise lessons to inform the development of the intervention to support primary or community healthcare professionals to provide advice rather than the substantive content of the advice itself.

The research programme utilised a person-based approach (PBA) [21]. The PBA provides a methodological approach for ensuring that the user of an intervention and their context is considered through all stages of development. It offers tools (such as the ‘table of planning’) to help plan and progress toward developing a theory [22]. The PBA approach involves the systematic investigation of the “*beliefs*,* activities*,* needs and situation of the people who will be using the intervention”* (p.1) through research [21]. Its aim is to “*ground the development of behaviour change interventions in a sensitive awareness of the perspective and lives of the people who will use them”* (p.1) [21]. This involves the development of ‘guiding principles’ which focus on what is needed to make the intervention acceptable, feasible, useful, and engaging for the users for whom it is designed. These are developed and progressively refined throughout a study as evidence and data are gathered [23]. Reflecting the PBA - the wider framework used for this study [21] - attention in the review was given to relevant contextual or environmental factors; influences on target behaviours; barriers and facilitators of the intervention; and promising intervention features.

This review is registered in the International Prospective Register of Systematic Reviews PROSPERO (CRD42022372456) and is reported according to PRISMA (Preferred Reporting Items for Systematic Reviews and Meta-Analyses) guidelines [24].

### Search strategy and selection criteria

The following five databases were searched separately: MEDLINE (Ovid); PsycINFO (Ovid); EMBASE (Ovid); Cumulative Index to Nursing and Allied Health Literature (CINAHL) (EBSCO); and Cochrane Central Register of Controlled Trials (CENTRAL). Concepts for the search strategy were: non-pharmacological interventions; healthcare professionals in primary or community settings; people living with dementia; and living at home/in the community. The search strategy and search terms were developed by the research team and reviewed and checked by King’s College London librarians. A search strategy was developed appropriate to each database. These are detailed in the supplementary tables (Additional File 1). Retrieved citations from each database search were imported into an Endnote library. Endnote’s automated de-duplication feature was used to remove duplicate references and the database was also manually checked for duplicates. Titles and abstracts of articles were screened separately by two researchers (HC and CM) against the agreed inclusion and exclusion criteria using a bespoke screening form. Full text copies were also screened by the same two reviewers (HC and CM). Endnote was used to manage references and Excel was used to record decisions on eligibility for inclusion. Disagreements were resolved through discussion between reviewers, consulting with a third researcher if necessary (BB).

Studies were selected according to the criteria in Table 1. The parameters for inclusion/exclusion included: language; study type; setting; participants; date of publication/data collection; type of intervention; intervention intensity; and publication type. Searches were restricted to articles published since the first national dementia strategy in England [25] with articles subsequently excluded if data collection pre-dated this. Searches were limited to the English language, but no geographical restrictions were applied. All empirical studies were included. As we were interested in interventions delivered *to* healthcare professionals (with the end goal of helping them to provide support) and interventions delivered *by* healthcare professionals to people living with dementia and their (paid and unpaid) carers, the population of interest included all these groups. As we were interested in interventions with a significant element of information but with a modest amount of face-to-face or other direct contact (as our proposed intervention was being designed to fit within the existing clinical environment), intervention intensity [26] was included as a parameter. Intervention intensity is defined as dose x dose frequency x total intervention duration. We limited the total intensity to no more than three hours of healthcare professional time. However, no restrictions were placed on number of online or email contacts or frequency/duration of self-directed learning. Studies were also restricted to those relating to the providing support with either IADLs (such as medication management and communication) and ADLs (such as mobility and help with going to the toilet) [27]. Due to the limited literature specifically focusing on ADLs, we additionally included interventions that may more broadly address supporting the management of behavioural symptoms supporting with/managing behavioural symptoms (e.g. apathy or indifference) aimed at enabling the person living with dementia to continue to perform their usual ADLs or the carer to provide support with them. Therefore, if the intervention focussed on supporting the management of behavioural symptoms it had to do so in the context of a carer providing daily care which, by its nature, is supporting ADLs/IADLs [5, 9, 28, 29]. This is because many of the continence challenges faced by carers are associated with behavioural problems (e.g. apathy or repetitive habits). For this reason the two Huis in Het Veld et al.’s publications [30, 31] were included as they included elements to manage apathy or indifference and nighttime restlessness.

**Table 1:** Inclusion and exclusion criteria

Parameters	Inclusion criteria	Exclusion criteria
**Participants/care recipient group**	• Healthcare professionals supporting people (18 years and over) living with dementia at home and their (unpaid and paid) carers • People with dementia (18 years and over) and their (unpaid and paid) carers	• Children/adolescents (under 18 years) • Healthcare professionals supporting child/adolescents (under 18 years) or adults with other conditions only • People with mild cognitive impairment
**Language**	English language	Language other than English
**Type of intervention**	Any non-pharmacological intervention^a^ involving primary or community healthcare professionals, to support the provision of management advice aimed at supporting people living at home with dementia and their (unpaid and paid) carers with activities of daily living ^b, c^	• Pharmacological intervention or use of supplements • Devices, technology or adaptations • Exercise or physical activity or participating in a specific activity (e.g. singing) • Therapy^d^ (e.g. counselling, cognitive stimulation/rehabilitation, occupational therapy) • Evaluation of model of care, specific role or service • Support groups or forums
**Intervention intensity** ^**e**^	Interventions with three or fewer face-to-face contacts of one-hour duration or less	Interventions with more than three face-to-face contacts or contacts of more than one-hour duration.
**Setting**	• Primary care • Community setting (own home or carers’ home)	• Care homes (residential and nursing) • Group homes • Sheltered/supported housing • Residential respite care • Residential rehabilitation • Day care or respite care outside the home • Hospital • Long-stay facilities
**Dates**	Data collected from 3^rd^ February 2009	Data collected before 3^rd^ February 2009
**Design/study type** ^**f**^	Empirical (quantitative and qualitative) work using both primary and secondary data and a variety of methodologies including quality improvement studies, case studies, national surveys and systematic reviews.	Commentaries, opinion pieces and descriptive articles without relevant empirical data. Observational studies without an intervention.
**Publication type**	Peer reviewed literature only	• Individual client case studies • Book reviews • Editorials • Study protocols • Commentaries/opinion articles • Conference abstracts • Dissertation/PhD theses • Non-peer reviewed literature • Grey literature (including research reports and national government reports)

^a^“*any sort of intervention not directly involving a medication; attempting to optimise a complex patient’s healthcare needs or to better manage their chronic illness”* [28]. Their aim is to *“improve or at least maintain the individual’s cognitive function*,* enable the person to continue to perform usual activities of daily living*,* and/or address behavioural symptoms that often accompany memory impairment”* [29].

^b^Includes interventions developed or delivered by healthcare professionals or delivered to healthcare professionals to help them to support people living with dementia and carers at home. The term paid carers refers to homecare workers.

^c^ [6, 7].

^d^Therapy includes: mindfulness, music therapy, reminiscence therapy, cognitive stimulation therapy/brain stimulation/cognitive rehabilitation, physical therapy or occupational therapy; counselling or emotional support; group therapy or support; acupuncture/acupressure.

^e^ Intervention intensity is defined as “dose x dose frequency x total intervention duration” [26]. We have limited intervention intensity to 3 h of healthcare professional time (maximum of 3 one-hour sessions).

^f^studies that reported findings rather than theoretical or conceptual pieces “*the simple test of relevance for inclusion is to specify that each reference must relate to some form of research*,* inquiry*,* investigation or study”* [32].

### Data extraction and analysis

Using a bespoke predesigned data extraction tool adapted from the Joanna Briggs Institute’s data extraction form [33], data were extracted by HC on study design and methodology; country; sample size including types of participants; development/delivery of the intervention and its components; and the focus of the intervention (reported in Table 2). Data relating to intervention development, implementation and evaluation were also extracted [34]. Within this, reflecting the wider framework used for this study [21], attention in the data extraction process was given to relevant contextual or environmental factors; influences on target behaviours; barriers and facilitators of the intervention; and promising intervention features by including these as elements on the data extraction form (Additional File 2).

**Table 2:** Summary of included studies

Reference	Country	Study design	Sample size	Intervention development/delivery	Components	IADL/ADL focus^c^	Themes^d^
Barry et al. (2020) [40]	Northern Ireland	Semi-structured interviews to inform its development	N = 30 (15 GPs; 15 community pharmacists)	Development of intervention for delivery by community pharmacists to PLWD living in the community	-Online video and quick reference guide for pharmacists including tips on communicating with PLWD and monitoring adherence -Face to face consultation with PLWD and carer	Medicines management including adherence	1, 3, 5 and 6
Cristancho-Lacroix et al. (2015) [41]	France	Pilot randomised controlled trial; semi-structured interviews at follow-up	N = 49 informal caregivers (intervention = 25; control = 24)	Evaluation of web-based psychoeducational program for informal caregivers – recruited by geriatricians and forum moderated by clinical psychologist	− 12 self-directed online sessions -theoretical and practical information, videos and practice guide – online forum for caregivers moderated by clinical psychologist	Caregiver skills to manage daily life difficulties (including strategies to facilitate performance of daily activities)	1, 2 and 3
Gaugler et al. (2015) [42]	USA	Pre/post-test design (survey)	N = 41 family caregivers	Evaluation of online psychoeducational intervention for family caregivers of PLWD including videos from community health professionals offering advice	-3 one-hour online training modules - content includes strategies to help individuals with dementia function independently and safely	Information and strategies to assist individuals living with dementia with performance of activities of daily living (including for example, mobility)	1, 2, 3, 4, 5 and 6
Gies and Pierce (2021) [43]	USA	Literature review, web-based survey and evaluation	*N* = 10 caregivers (survey) *N* = 12 caregivers (evaluation)	Development of web-based educational modules developed by nursing professionals/students for homecare family caregivers and also intended to be used (and added to in the future) by healthcare nurses and homecare clinicians	6 web-based educational modules (gender-specific)	Information and strategies to educate carers when assisting with activities daily living including information about how to communicate and interact when care giving	1, 2, 3, 4 and 6
Huis In Het Veld et al. (2019)^a^ [30]	Netherlands	Mixed-methods process evaluation alongside RCT (including analysis of email contact and email/website analytics)	Semi-structured interviews: *n* = 12 caregivers Interventionists (dementia nurses) *n* = 4 Survey: *N* = 81 caregivers	Process evaluation of online self-management support intervention for family caregivers including contact with dementia nurse	Three arms: 1) Major self-management support intervention including email contact with specialist dementia nurse and online videos and e-bulletins 2) online videos and e-bulletins 3) e-bulletins Information and tailored care giving strategies	Support family caregivers to manage behaviour changes to maintain caring relationship, promote better interactions, self-management of daily problems relating to care recipient’s dementia and perceived competence in caring for someone with dementia	1, 2, 3 and 6
Huis In Het Veld et al. (2020) [31]	Netherlands	3- arm randomised control trial	*N* = 81 caregivers of people living with dementia at home	Evaluation of online self-management support intervention for family caregivers including contact with dementia nurse	Three arms: 1) Major self management support intervention including email contact with specialist dementia nurse and online videos and e-bulletins 2) online videos and e-bulletins 3) e-bulletins Information and tailored care giving strategies	Support family caregivers to manage behaviour changes to maintain caring relationship, promote better interactions, self-management of daily behaviour problems relating to care recipient’s dementia and perceived competence in caring for someone	1, 2, 3 and 6
Lewis et al. (2010) [44]	USA	Formative evaluation (participation in program and follow-up questionnaire)	*N* = 47 caregivers	Evaluation of internet-based psycho-educational program for caregivers of people with dementia developed by professionals and caregivers	Protype of 4 online modules from 18 core modules of the existing Savvy Caregiver Program	Provide carers with knowledge and skills they need to assist with activities of daily living	1, 2, 3 and 5
Metcalfe et al. (2019) [45]	Germany, France, England	Pilot randomised controlled trial; semi-structured interviews	*N* = 61 caregivers	Evaluation of online information and support programme for caregivers of individuals diagnosed with young onset dementia. Including content provided by professionals.	7 modules in multimedia format combining written and video content, case-studies, presentations from professionals, and downloadable materials.	Information and skills-building of caregivers supporting with activities of daily living and information about available care and support and common problems and solutions	1, 2 and 3
Meyer et al. (2016) [46]	Australia	Semi-structured interviews (baseline and follow-up)	*N* = 25 dyads (caregivers and people living with dementia)	Development of individualised falls prevention intervention delivered by community healthcare professionals	- assessment tools and discussion tool to identify and rank falls risk factors - provide options for falls prevention strategies	Education and advice to balance/reduce risk of falls whilst supporting the person living with dementia, maintaining independence and activity levels	2, 3, 4 and 6
van der Roest et al. (2010)^b^ [38]	Netherlands	Pilot study (pretest–posttest control group design).	*N* = 28 informal caregivers (intervention *n* = 14; control *n* = 14)	Development of DEMentia-specific dynamic interactive social chart (DEM-DISC) aimed at carers, people with dementia and professionals.	-Information on practical support and coping -General and tailored information on dementia care and welfare services that could potentially fulfil needs	Information about dementia, associated needs, and care and support services to help support the person living with dementia with activities of daily living	1, 2, 3 and 5
Van Mierlo et al. (2015) [39]	Netherlands	Cluster randomised trial ; semi-structured stakeholder interviews	*N* = 73 caregivers (intervention *n* = 41; control *n* = 32) *N* = 19 case managers	DEMentia Digital Interactive Social Chart (DEM-DISC) an ICT tool to support customized disease management in dementia for use by case managers and family and professional caregivers	-Information on practical support and coping -General and tailored information on dementia care and welfare services that could potentially fulfil needs	Information about dementia, associated needs, and care and support services to help support the person living with dementia with activities of daily living	1, 2, 3, 4, 5 and 6
Yates et al. (2019) [47]	United Kingdom	Analyses of two longitudinal databases of older adults Interviews with older people, people living with dementia, and carers Consensus expert review	*N* = 32 PLWD* n* = 4 Carers *n* = 11 Dementia advisors *n* = 14 Older people *n* = 3	Post diagnostic social intervention to help people living with dementia to live well and as independently as possible. Manual based intervention for delivery by health and voluntary sector professionals	3 core topics and 7 optional topics 3 sessions with dementia adviser who helps dyad plan activities, identify resources and signpost to resources that might be useful, review plans, and adjust them	Support people living with dementia to self-manage their care, continue with or enhance involvement in activities and make everyday decisions	2, 3, 4, 5 and 6

^a^This was a process evaluation which accompanied the RCT [31]; ^b^This related to an earlier stage of development of another intervention [39]; ^c^IADL (Instrumental Activities of Daily Living) and Activities of Daily Living (ADL);^d^Themes from the literature were: (1) Mode of delivery; (2) targeted or tailored resources; (3) content, design and navigation; (4) credibility of the information or resource; (5) user involvement in development and evaluation; and (6) role of professionals and organisations

One reviewer (HC) assessed the quality of included empirical papers using the Mixed-Methods Appraisal Tool (MMAT) [35]. This was used as the review covers a range of studies including qualitative research; randomised controlled trials; non-randomised studies; quantitative descriptive studies; and mixed-methods studies. These judgements were checked by a second reviewer (CM) with disagreements resolved through discussion. This was undertaken for descriptive purposes and did not form part of the inclusion criteria.

Given the purpose of the review and that the studies had a variety of objectives and research designs, a narrative synthesis approach was adopted [36]. This is the *“systematic review and synthesis of findings from multiple studies that relies primarily on the use of words and text to summarise and explain the findings of the synthesis …. the defining characteristic is that it adopts a textual approach to the process of synthesis to ‘tell the story’ of the findings from the included studies”* (p.5) [36]. Particularly relevant to this review is its suitability for systematic reviews focussing on a wide range of questions, not just those relating to the effectiveness of interventions. Here, information about the development and delivery of interventions, including details of barriers and facilitators of interventions, were the main outcome of interest rather than specific outcomes of an intervention or study. Extracted data from each article were collated and themes were identified by HC and then discussed with co-authors, stakeholders, and public contributors. A narrative encompassing these themes was then developed to synthesize and interpret the evidence from the systematic review to address the purpose and bring together evidence to inform the development and evaluation of the intervention being developed by the research team.

Thus this review incorporated the following main broad elements of a narrative synthesis process identified by Popay et al. [37]: developing a theory of how the intervention might work, why and for whom (by using the person-based approach as our framework to inform our data extraction); developing a preliminary synthesis of findings of included studies (an initial description of the results of included studies – Table 2); exploring relationships in the data (by synthesising the findings and identifying themes across the studies, with a focus on intervention facilitators and barriers – narrative synthesis under theme headings); and assessing the robustness of the synthesis (incorporation of systematic searches, quality appraisal as above and reflections on the quality and quantity of available literature). The findings from the review were then combined with those from qualitative interviews and stakeholder feedback using a Table of Planning (a tool used in the PBA) to work towards developing a theory of how the proposed intervention might work [22].

## Results

### Selected studies

**Fig. 1:**
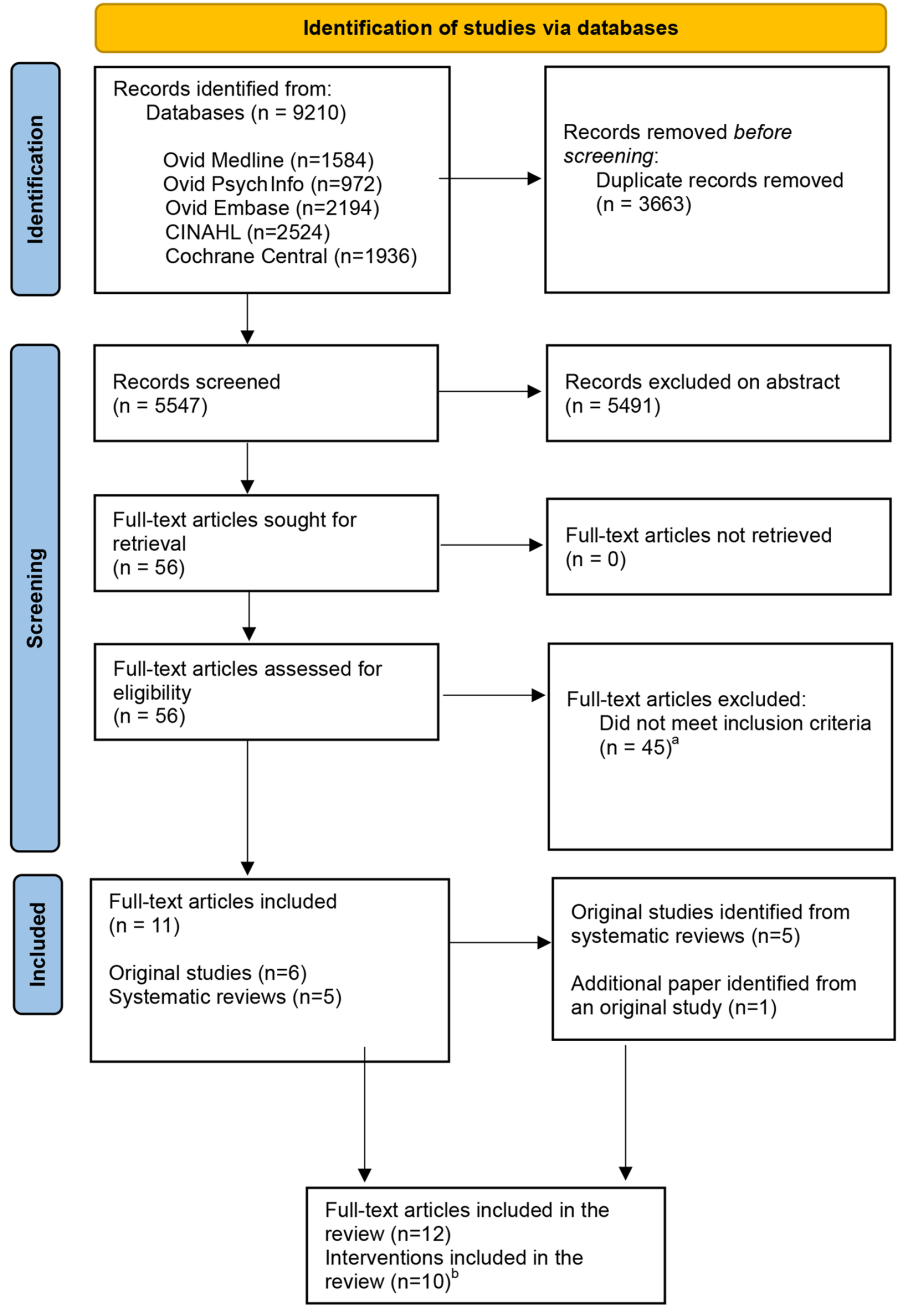
PRISMA flow diagram of studies included in the review. ^a^Reasons: Intensity (*n* = 10); literature review with no relevant articles (*n* = 9); not focussed on activities of daily living (*n* = 8); therapies (*n* = 5); data collected before 3rd February 2009 (*n* = 5); not delivered to/by (or developed by) healthcare professionals (*n* = 3); devices, technologies or adaptations (*n* = 2); not in primary care or community setting (*n* = 1); observational study without an intervention (*n* = 1); and physical activity (*n* = 1). ^b^two sets of two articles related to the same intervention. One of these was a process evaluation [30] which accompanied the Randomised Control Trial [31] and one [38] related to an earlier stage of development of another [39]

Of 5547 unique references identified through searches, 56 were selected for full text assessment from which 11 papers were eligible for inclusion in this review (Fig. 1). One additional paper was included which provided details of a process evaluation [30] undertaken as part of one of the included studies [31]. We also included two papers related to the same intervention at different stages of development [38, 39]. This resulted in 12 papers reporting on 10 interventions being included.

Table 2 provides an overview of the included studies. Most of the 12 papers were from The Netherlands (3) or the United States (US) (3). Other countries represented included Australia (2); France (1); Northern Ireland only (1); and United Kingdom (1) with one multi-country study (Germany, France, England). Most interventions (8 of 10) focussed on general information, knowledge, skills or strategies to support people living with dementia with activities of daily living. However, 2 had a more specific focus (1 medication management and adherence; 1 strategies to reduce the risks of falls at home). Half (5 of 10) of the interventions comprised online resources; 2 online resources and face-to-face contact with a professional; 1 online resources and email contact with a professional; 1 online resources and contact with other carers through a forum (moderated by a professional); and 1 face-to-face contact only. A variety of methodologies was utilised amongst the 12 papers: 4 randomised controlled trials (2 pilot, 1 three-arm and 1 cluster); 2 pre-test/post-test designs; 2 qualitative studies (semi-structured interviews); 2 surveys/questionnaires; 1 study utilising longitudinal and interview data; and 1 process evaluation. The quality of included studies as measured using MMAT was variable with scores ranging from 0 to 5 (with an average of 2). However, as recommended by Hong et al. [35]. , no study was excluded on quality grounds.

What follows is a narrative review organised by themes identified in the literature. These themes, including subthemes, are summarised in Table 3. These six themes were: mode of delivery (including the value of online interventions and personal contact); targeted or tailored resources; content, design, and navigation; credibility of the information or resource; user involvement in development and evaluation; and role of professionals and organisations.

**Table 3:** Summary of themes from the literature

Theme and subthemes	Description
1. Mode of delivery	Method used to deliver the intervention.
a. Value of online interventions	Value of online interventions including removal of geographical barriers, need for travel and their continuing availability to the carer and others who may be involved in caring for the person living with dementia.
b. Personal contact	Personal contact with professionals or carers in similar situations including feedback about caring strategies and someone to listen and discuss problems with.
2. Targeted and tailored resources	Targeting and tailoring resources to ensure relevance and they reach those that need them. Includes considerations such as the different stages of dementia, the importance of delivering information at an appropriate level and time and taking account of existing knowledge, characteristics, experiences and preferences of people living with dementia and their carers.
3. Content, design and navigation	The information and its presentation
a. Content	What information was made available including links to other sources of information. Includes the amount of information provided, its ordering and level of detail.
b. Style, presentation and design of information	Manner or style in which information is displayed and look and function of websites. Importance of good information being carefully presented in a choice of formats.
c. Tone and readability	Choice of words and how the text makes people feel when reading it. Information should not be presented in a patronising or insensitive way. The educational and reading level of potential readers needs to be considered so that information is easy to understand and read.
d. Navigation	User friendliness and ease of use are important so that written material or website are easy to navigate. Navigation instructions should be clear and without onerous steps.
4. Credibility of the information or resource	Trusted up-to-date source of information from people knowledgeable about the subject, underpinned by an appropriate and quality evidence base.
5. User involvement in development and evaluation	User involvement to ensure relevance and utility of information and user-friendliness of the chosen mode of delivery.
6. Role of professionals and organisations	Role for professionals in referring people to information and helping them navigate and apply it to their own circumstances. Support of professionals and organisations in terms of keeping content relevant, up-to-date, and accessible in the longer term.

### Mode of delivery

The mode of delivery, that is the method used to deliver the intervention was a significant theme. Within this, two sub-themes emerged relating to the value of online interventions (relative to in-person delivery) and personal contact either with professionals, or carers in similar situations.

### Value of online interventions

The value of online interventions was a significant theme in the literature [30, 31, 38, 40, 42–45]. In particular, studies noted how making information resources available online removed geographical barriers and the need for carers to travel, thus beneficial for those who are time poor or who cannot leave the person they are supporting [31–38, 42–45]. Online resources can be accessed nationally or internationally at carers’ own convenience without incurring travel expenses or disrupting work or home life [31, 38, 43] if they have online access and are digitally confident. Metcalfe et al. [45] highlighted that “*the true value of an online resource may be through its continuing availability . being able to ‘dip in and out’ of the resource as and when information is needed”* (p. 1462). This was also echoed by Gaugler et al. [42] who also noted that carers valued being able to share this information with other family members to help educate them about caring. A sense of control over when and where resources are accessed and for how long was identified as beneficial for carers [44] and online availability of information was perceived as making this more accessible to healthcare professionals working in busy clinical settings [40].

Nevertheless, it was also noted that online interventions can sometimes create barriers to accessing information resources and it is important to minimise these wherever possible, for example, by minimising or excluding the use of usernames and passwords to access information or systems [39]. There may also be different barriers to accessing resources depending on the age and relationship of the carer to the person living with dementia. For example, whilst adult child carers may be time poor even if they might value access to online resources, spouses may have more difficulty accessing computers or the internet [39]. There can also be technical problems with accessing, navigating, or using content including the impact of poor internet connections or availability of appropriate software [38, 41, 42]. In terms of developing and evaluating online resources, it is important to recognise that the need for computer-skills may lead to selection bias of those included within such studies [38].

### Personal contact

There was a suggestion in the literature that carers would have liked more human interaction with professionals or peers including face-to-face or email contact [30, 31, 41, 44, 45]. Gaining personal feedback via email regarding how they were caring for their relative and recognition of their caring role was particularly valued [30]. From the perspective of healthcare professionals, they felt that family carers “*were looking for a release valve and a listening ear”* (p.8) [30]. However, this research also suggested that email contact may not suit all carers, with phone contact reportedly being preferred by them, either because of lack of time or because of the difficulty of putting emotions and questions down on ‘paper’. Nevertheless, one healthcare professional in the same study believed that putting the situation down on paper might help give family carers a better picture of the situation [30]. However, when an online forum was made available in another study it was not well-used, which might suggest other (less public) means of communicating with others may be more effective [41].

### Targeted and tailored resources

The targeting and tailoring of resources were viewed as important to ensure relevance, uptake and those that need the information or support the most receive it [30, 31, 38, 39, 41–44, 46, 47]. Particularly important in this context was having an awareness of the different stages of dementia, where people may be in their dementia ‘journey’ (for example, have they just been diagnosed), and that their needs and information requirements will change over time. Information about the different stages of dementia and what to anticipate as the disease progresses is viewed as important [42] but professionals may also need to be mindful of the plethora of information sometimes given at the time of the diagnosis and avoid information overload [46]. Thus, it was considered important that information is provided at an appropriate level, in an appropriate way at an appropriate time [46] so that people are prepared but are not made to feel anxious about what may happen in future [41, 46]. This is also important as the perceived relevance of information by carers has been reported to depend on the stage of dementia their relative had reached [41, 45]. Therefore, information should be provided in a timely and appropriate manner, to ensure personal relevance and to avoid being patronizing or anxiety provoking [30, 46].

Also prominent in the literature was the need to tailor information to individual needs and preferences and to have an awareness or sensitivity to the existing knowledge, experience and preferences of people living with dementia and their carers, the context of their lives, and how this may impact on their uptake of information or strategies [46]. Awareness of a person’s educational and work background is required [41, 47]. For effective knowledge translation, it is important to respect individuals and help them to retain their sense of agency, they are *“more than just empty vessels to be filled”* (p.7) [46]. Having details of carers’ personal characteristics and contextual information may help to customise information and recommendations to their personal situation [39]. It is also considered important to review materials to ensure they are culturally appropriate [44]. Studies have also suggested that the utility of information may be assessed differently depending on both the gender of the carer and their relationship to the person living with dementia [41, 43].

### Content, design and navigation

The most significant theme in the literature was the importance of attention to the content, design and navigation of websites or resources. Lessons learned focussed on content (what information was made available); style and presentation of information (manner or style in which it was displayed) and design of websites (look and function); tone (choice of words used, how it made people feel when reading them) and readability (how easy it was to understand and read); and navigation (how easy the material or website was to navigate).

#### Content

In terms of topics covered, carers were reported to prefer content regarding caring for their friend or relative and to be less interested in that relating to self-care, that is caring for themselves as a carer [41]. Information about the different stages of dementia and what to expect as it progresses is viewed as important by carers [42]. Having ‘real’ individuals represented in video content was also valued by carers, who found it comforting to hear other people’s stories which made them believe that they could cope with caring challenges and to understand and relate to the information presented [30, 31, 42, 45]. Professionals also valued video content demonstrating key intervention behaviours and including positive feedback about their outcomes from professionals, carers, and people living with dementia [40]. Links to other resources or information were viewed as helpful by both carers and professionals [39, 40]. Information needed to be in sufficient depth, but a balance needed to be struck between the level of detail and the amount of text included on pages [38, 42, 44]. The order information was presented in was also important, with evidence of topics being presented later being visited less often [45]. Also how resources end was important, with one study suggesting they should end with an uplifting message for carers, such as that they are able to make the best of a difficult situation [42].

#### Style, presentation and design

With regards to style, presentation, and design there was a sense that good information needed to be attractively and carefully presented [40, 43, 44, 47]. Information overload should be prevented by breaking material by sections and avoiding repetition [44, 46]. Large font sizes should be used and designs should be free of clutter and distracting design features [43, 47]. Quality of presentations should be ensured by multiple recording sessions where needed [43]. Materials needed to be viewable on mobile devices [43], to be printable as a workbook [44], or used as a quick reference guide [40] so that they can be easily accessed in a suitable format when needed.

#### Tone and readability

The tone and readability of the website or material were also considered important. Information should be presented accessibly, considering the educational and reading level of possible readers and different levels of understanding, whilst avoiding being patronising [43, 44, 47]. Topics should also be approached sensitively with an awareness of subjects that might risk upsetting people to talk or think about, for example the lack of a social network [47].

#### Navigation

With regards to the navigation of websites or resources, user friendliness and ease of use were important, such as ensuring navigation instructions were clear and material accessible without too many steps or mouse clicks [39, 42–44, 47]. For example, Gies and Pierce [43], in response to feedback from users, made all modules accessible on one page using one mouse click. Being able to easily dip in and out of resources and being able to identify which sections had already been visited (for example, through using an automated tick) was considered important [44]. Having clear indexing of content and search functions were regarded as helpful in terms of enabling carers to identify topics of most interest and, importantly, to avoid those they may find unhelpful or do not yet want to read [45].

### Credibility of the information or resource

Another significant theme was around the importance of the intervention being a trusted source of information and key to this was users being able to easily assess the credibility of the information provided [43, 46]. For example, Gies and Pierce [43] noted the importance of writer and organisation credentials being clear and easy to identify on the website. Similarly, all content including text, video, audio, and graphic content should be underpinned by an evidence base, which also not only increases credibility, but the likelihood that it is appropriate for the behaviour it seeks to target, and its chance of being used and effective [42, 47]. Contributing to the credibility of a resource is ensuring that content, references and weblinks are kept up-to-date [39]. Furthermore, in terms of its credibility, information needs to be provided or delivered by someone knowledgeable about the subject [46].

### User involvement in development and evaluation

Studies highlighted the importance of involving people living with dementia, carers and professionals from an early stage in the development and evaluation of websites or resources to ensure user-friendliness [38–40, 42, 44, 47]. In Gaugler et al.’s [42] study, an expert panel of clinical and scientific experts on family caregiving and family carers identified relevant content and developed and evaluated prototype modules. Lewis et al. [44] developed the content and presentation of their intervention in an iterative process in response to feedback from experts in dementia caregiving and family carers. Another study undertook qualitative interviews with healthcare professionals, people living with dementia and carers to inform intervention development [40]. This involvement was seen as helpful in ensuring that the intervention addressed matters of importance to the end user and were relevant to and applicable in daily practice. It was also thought to contribute to a better chance of the intervention benefiting the target population and of successful implementation and uptake. However, in a cautionary note, they added that those most likely to engage with the development and evaluation of interventions may be those with a strong interest in or awareness of the subject matter who may not necessarily be representative of those being targeted to use the intervention in practice.

### Role of professionals and organisations

The role of professionals, including healthcare professionals, emerged as a significant theme in the studies reviewed. More specifically, the importance of their role in signposting or referring people to the information or helping them to navigate it was addressed [30, 39, 46, 47]. Meyer et al. [46] referred to the idea of a *“knowledge broker acting as a channel through which to connect health-care resources and information “at the right time*,*” thus sustaining the dyad in their role as long as is feasible”* (p.9). They emphasised the important role of professionals in identifying problems and providing individualised advice and strategies. Yates et al. [47] described how a manualised approach to delivering interventions with the involvement of professionals/intervention provider enables both standardisation of delivery and personalisation of content and the communication of information in an accessible and appropriate way through discussion. Another study cited the need to raise support among professionals who can direct carers to the information [39]. Huis In Het Veld et al. [30, 31] highlighted the role of healthcare professionals in helping carers to translate information and advice to their own situations. They recommended ensuring healthcare professionals who are delivering the intervention are clear about their role in the intervention and the importance of integrated use of intervention elements, where applicable.

The support of professionals and organisations was also viewed as important in terms of keeping content relevant and up-to-date and gaining funding to keep it going long-term [39, 43]. In terms of getting this support, recommendations included involving new organisations which need to profile themselves who may be motivated to get involved for this reason. To engage both individuals and organisations, those developing and implementing interventions need to show how they fit with current policy developments so that this gets priority when there are multiple other competing priorities and staff have high workloads [39, 40]. Another way of gaining support or interest is emphasising to the value of such interventions in situations where people are receiving routine clinical care but time to address carers’ concerns is limited [42].

## Discussion

The aim of this review was to identify and synthesise lessons learned from available literature on interventions, involving healthcare professionals in primary or community settings, to support the provision of management advice aimed at supporting people living at home with dementia and their carers with ADLs. This included but was not limited to toileting or continence care. It was undertaken to inform the development and implementation of an intervention to support healthcare professionals to proactively provide existing continence management advice to the carers of people living at home with dementia. A total of 12 articles, reporting on 10 studies were included. A strength of this review is its novel nature, to the authors’ knowledge it is the first systematic review to focus solely on this specific type of intervention. Exhaustive literature searches and rigorous eligibility criteria and data extraction processes led to the selection of relevant articles with a tight focus that enabled careful teasing out of valuable insights into matters to consider in the development and implementation of this type of intervention. Whilst the search was systematic, the synthesis of the results was limited to a narrative synthesis. As the focus of the review was not the effectiveness of interventions, but on their development, delivery, and evaluation and, because reviewed studies had a variety of methodologies, a narrative synthesis was entirely appropriate [36]. A limitation of the findings is that the number of included studies is small and the methodological quality of most studies was moderate. Moreover, although no geographical restrictions were placed on the inclusion of studies, only studies published in English were included which may have limited our review. Furthermore, due to the lack of continence-specific interventions, it was beneficial to translate lessons from interventions that were not specifically focussed on continence. Nevertheless these provided useful insights into the delivery of practical advice to carers supporting people living with dementia to manage day to day activities.

The present review highlighted several important considerations and lessons for the development of our intervention. Themes were identified from the literature including the mode of delivery (such as the value of online interventions and personal contact); targeted or tailored resources; and content, design and navigation. Information for carers has been noted to be often sufficient in quantity but less frequently designed to accommodate their access needs. Moreover, lack of timeliness, relevance, and personalisation of the information can mean that key information needs remain unsatisfied [48]. Present, but less prominent in the literature, was a theme around the credibility of information and, importantly, how this can be ensured and maintained in the longer term so that carers feel it is trust-worthy. In the broader literature, Rowley et al. [49] concluded that a key factor influencing people’s trust in information is it being perceived as credible and accurate. Carers have reported feeling they have a duty to ensure they access trustworthy and reliable information to inform the care they give to their relative [48]. In this context, it was important to carers that the creator of the information was well recognised and knowledgeable, they particularly viewed information provided by ‘official’ organisations such as the National Health Service (NHS) and the Alzheimer’s Society as being sources of quality, trustworthy and reliable information. In contrast, commercial websites were viewed as untrustworthy and motivated by profit rather than genuine support [48]. Other studies outside of this review have also reported that carers particularly value information and advice provided by carers with firsthand experience of caring and healthcare professionals as they are considered trustworthy sources of evidence-based information advice that has been tested by other people [48, 50, 51]. Knowing that other carers are going through similar situations, has been reported to help reduce feelings of isolation and helplessness while delivery of practical advice by healthcare professionals may also have positive benefits in terms of promoting trust in healthcare professionals more generally [48, 52]. These findings were echoed by interviewees, stakeholders and public contributors in the current programme of research [10, 14, 20]. Overall, these findings suggest that making it clear how carers and professionals have contributed to the development of resources is important in terms of ensuring that carers accept their credibility and trustworthiness.

However, importantly, compared to other themes identified in the literature, less was written about the role of healthcare professionals and user involvement in the development and evaluation of these resources. This may have important implications in terms of the perceived credibility of information resources and interventions because, as noted above, carers are more likely to trust information provided by healthcare professionals and other carers but less likely to trust information where they cannot identify its origins or assess its trustworthiness. Generally, a lack of detail around this within the studies reviewed presented a challenge in the screening and data extraction process for this review. Nevertheless, what emerged strongly in the reviewed literature was the importance of healthcare professionals in signposting people to information or helping to navigate it. This is significant as other studies have described how carers can sometimes find it difficult to locate, access and navigate authoritative and reliable information and that potentially healthcare professionals could play a vital role in assisting carers in this task [48, 53]. Other research has also pointed to the value of having a central source of online information on a specific medical condition, providing free and quality-assured information for patients, carers and healthcare professionals to access [54]. Moreover, Harrop et al. [55] suggested that healthcare professionals, in their routine interactions with patients and their families, could more explicitly encourage requests for help and advice from relatives and combine this with providing an information pack. They argued not only would this *“legitimate given topics as valid areas for discussion”* (p.8) and *“give carers a sense of legitimacy as credible seekers of information*,* help and support”* (p.8), but also in the longer term this might lead to considerable cost savings to the public purse associated with preventing family care breakdowns.

Whilst studies included within this review were very informative, this review has revealed a dearth of studies to inform the development, delivery, and evaluation of interventions to support the provision of management advice aimed at supporting people living with dementia at home and carers with ADLs. The researchers involved in the literature search and review process noted that there were fewer studies relating to people living with dementia in the community compared to care homes. Also many of these studies were testing specific therapies (for example, counselling or occupational therapy), exercise/physical activity interventions, use of devices or technology or interventions focussing on carers’ self-care. Moreover, by comparison, there were very few studies relating to the efficient provision of practical information, education, training or advice that can engage healthcare professionals, paid and family carers to help them to support people living with dementia at home with ADLs. Furthermore, where studies do exist often the actual intervention, its development, and delivery are not well described which can make screening and extracting data from these studies challenging. For these reasons, we elected to keep our interpretation of interventions designed to provide management advice aimed at supporting people living at home with dementia and their carers with ADLs broad to enable the inclusion of papers where the intervention was aimed at supporting self-management and behavioural problems to indirectly support daily activities (for example Huis In Het Veld et al.) [30, 31].

More generally, there is scant empirical evidence evaluating the information needs of carers of people living with dementia [48]. Different populations of carers and their relatives or friends requiring care or support, including underserved or underrepresented populations, often need help to obtain timely and accurate online information that meets their needs [56]. However, carers’ specific information needs often go unmet, for example with regards to physical aspects of care such as, for example, hygiene assistance [55]. Thus, this review, has revealed considerable scope for more research in this area. Future research should ensure that interventions and their development are adequately described (for example, by utilising checklists developed for this purpose [57] as should the nature and extent of involvement of healthcare professionals, people living with dementia and carers. Not only is this important to ensure users are assured of the credibility of interventions, but it also enables other researchers to draw lessons from this research and apply these to their own studies.

In summary, this systematic review has identified several lessons which are helpful to the development of this and similar interventions. In the current broader study, these lessons were integrated with findings from qualitative interviews [20] and our discussions with public contributors and stakeholders using a person-based approach to intervention development [21]. The findings were combined in a “Table of Planning” to develop theory of how the planned intervention might work, why and for whom [22]. Together these findings are informing the development and delivery of an intervention to support healthcare professionals to provide continence management advice to the carers of people living at home with dementia [14]. Not only are the lessons relevant to the development of this intervention, they (summarised in Table 3) could constitute a valuable checklist for others to consider when developing and evaluating similar interventions.

## Conclusions

Despite the urgent need to better support people living at home with dementia, this review highlights the paucity of published studies reporting on interventions involving primary and community healthcare professionals to support the provision of management advice aimed at supporting this population with ADLs.

Nevertheless, this review has highlighted important considerations that have the potential to aid the development, delivery, and evaluation of the proposed and any future similar interventions. Future studies should provide more specific details about the development of interventions, in particular the involvement of people living with dementia, carers and healthcare professionals. This would enhance the credibility of interventions and enable others to benefit from lessons regarding the strengths and limitations of different approaches to user, carer and professional involvement.

## Electronic supplementary material

Below is the link to the electronic supplementary material.

Supplementary Material 1

Supplementary Material 2

## Data Availability

The datasets used and/or analysed during the current study are available from the corresponding author on reasonable request.

## Abbreviations

CENTRAL: Cochrane Central Register of Controlled Trials
CINAHL: Cumulative Index to Nursing and Allied Health Literature
MMAT: Mixed-Methods Appraisal Tool
PRISMA: Preferred Reporting Items for Systematic Reviews and Meta-Analyses
UK: United Kingdom

## References

[1] World Health Organisation. Dementia factsheet. World Health Organisation. 2023. https://www.who.int/news-room/fact-sheets/detail/dementia. Accessed 18 July 2024.

[2] Social Care Institute for Excellence (SCIE). Dementia: at a glance. SCIE. 2020. https://www.scie.org.uk/dementia/about/. Accessed 18 July 2024.

[3] House of Commons. Health and Social Care Committee Supporting people with dementia and their carers. Seventh Report of Session 2021–22 report. London: House of Commons; 2021.

[4] Alzheimer’s Society. Carers UK’s State of Caring 2021 report – Alzheimer’s Society responds. London: Alzheimer’s Society. 2021. https://www.alzheimers.org.uk/news/2021-11-03/carers-uks-state-caring-2021-report-alzheimers-society-responds. Accessed 18 July 2024.

[5] The King’s Fund. Caring in a complex world: perspectives from unpaid carers and the organisations that support them. London; The King’s Fund; 2023.

[6] Bucks R, Ashworth D, Wilcock G, Siegfried K. Assessment of activities of daily living in dementia: development of the Bristol activities of Daily Living Scale. Age Ageing. 1996;25(2):113–20. 8670538 10.1093/ageing/25.2.113

[7] Giebel C, Sutcliffe C, Challis D. Activities of daily living and quality of life across different stages of dementia: a UK study. Aging Ment Health. 2015;19(1):63–71. 24831511 10.1080/13607863.2014.915920

[8] World Health Organisation. Global action plan on the public health response to dementia 2017–2025. Geneva: World Health Organisation; 2017.

[9] Norton L, Malloy P, Salloway S. The impact of behavioral symptoms on activities of daily living in patients with dementia. Am J Geriatr Psychiatry. 2001;9(1):41–8. 11156751

[10] Murphy C, de Laine C, Macaulay M, Avery M, Fader M. A qualitative study and preliminary model of living with dementia and incontinence at home: beyond containment. Age Ageing. 2022;51(1):1–9. 10.1093/ageing/afab221 PMC8753012 34888621

[11] Drennan V, Cole L, Iliffe S. A taboo within a stigma? A qualitative study of managing incontinence with people with dementia living at home. BMC Geriatr. 2011;11:75. 22081876 10.1186/1471-2318-11-75 PMC3250935

[12] Schluter P, Ward C, Arnold E, Scrase R, Jamieson H. Urinary incontinence, but not fecal incontinence, is a risk factor for admission to aged residential care of older persons in New Zealand. Neurourol Urodyn. 2017;36(6):1588–95. 27778373 10.1002/nau.23160

[13] Hauber A, Mohamed A, Johnson F, Cook M, Arrighi H, Zhang J, Grundman M. Understanding the relative importance of preserving functional abilities in Alzheimer’s disease in the United States and Germany. Qual Life Res. 2014;23(6):1813–21. 24448684 10.1007/s11136-013-0620-5

[14] Murphy C, Chester H, Bradbury B, Morrison L, Manthorpe J, Fader M, Santer M, Ward J. Continence care for people living with dementia: a strategic priority. J Dement Care. 2022;30(6):18–9.

[15] Murphy C, De Laine C, Macaulay M, Hislop Lennie K, Fader M. Problems faced by people living at home with dementia and incontinence: causes, consequences and potential solutions. Age Ageing. 2021;50(3):944–54. 10.1093/ageing/afaa262. 33320926 10.1093/ageing/afaa262

[16] Richards D, Hilli A, Pentecost C, Goodwin V, Frost J. Fundamental nursing care: a systematic review of the evidence on the effect of nursing care interventions for nutrition, elimination, mobility and hygiene. J Clin Nurs. 2018;27(11–12):2179–88. 29156087 10.1111/jocn.14150 PMC6001513

[17] Cremer S, Vluggen S, de Man-Van-Ginkel J, Metzelthin S, Zwakhalen S, Bleijlevens M. Effective nursing interventions in ADL care affecting independence and comfort - a systematic review. Geriatr Nurs. 2023;52:73–90. 37269607 10.1016/j.gerinurse.2023.04.015

[18] Dawson A, Bowes A, Kelly F, Velzke K, Ward R. Evidence of what works to support and sustain care at home for people with dementia: a literature review with a systematic approach. BMC Geriatr. 2015;15:59. 25967742 10.1186/s12877-015-0053-9 PMC4465454

[19] Cheng S, Lin D, Hu T, Cao L, Liao H, Mou X, Zhang Q, Liu J, Wu T. Association of urinary incontinence and depression or anxiety: a meta-analysis. J Int Med Res. 2020;48(6). 10.1177/0300060520931348. 10.1177/0300060520931348 PMC7303787 32552169

[20] Bradbury B, Chester H, Santer M, Morrison L, Fader M, Ward J, Manthorpe J, Murphy C. Healthcare professionals’ experiences and views of providing continence support and advice for people living at home with dementia in England: that’s a carer’s job. BMC Geriatr. 2024;24(213). 10.1186/s12877-024-04830-8. 10.1186/s12877-024-04830-8 PMC10905774 38424477

[21] Yardley L, Morrison L, Bradbury K, Muller I. The person-based approach to intervention development: application to digital health-related behaviour change interventions. J Med Internet Res. 2015;17(1):e30. 25639757 10.2196/jmir.4055 PMC4327440

[22] Essery R, Pollet S, Smith K, Mowbray F, Slodkowska-Barabasz J, Denison-Day J, Hayter V, Bradbury K, Grey E, Western M, Milton A, Hunter C, Ferrey A, Müller A, Stuart B, Mutrie N, Griffin S, Kendrick T, Brooker H, Gudgin B, Phillips R, Stokes T, Niven J, Little P, Yardley L. Planning and optimising a digital intervention to protect older adults’ cognitive health. Pilot Feasibility Stud. 2021;7(158). 10.1186/s40814-021-00884-2. 10.1186/s40814-021-00884-2 PMC8371874 34407886

[23] Band R, Bradbury K, Morton K, May C, Michie S, Mair F, Murray E, McManus R, Little P, Yardley L. Intervention planning for a digital intervention for self-management of hypertension: a theory-, evidence- and person-based approach. Implement Sci. 2017;12(25). 10.1186/s13012-017-0553-4. 10.1186/s13012-017-0553-4 PMC5324312 28231840

[24] Page M, McKenzie J, Bossuyt P, Boutron I, Hoffmann T, Mulrow C, Shamseer L, Tetzlaff J, Akl E, Brennan S, Chou R, Glanville J, Grimshaw J, Hróbjartsson A, Lalu M, Li T, Loder E, Mayo-Wilson E, McDonald S, McGuinness L, Stewart L, Thomas J, Tricco A, Welch V, Whiting P. Moher, D. The PRISMA 2020 statement: an updated guideline for reporting systematic reviews. PLoS Med. 2021;18(3):e1003583. 33780438 10.1371/journal.pmed.1003583 PMC8007028

[25] Department of Health. Living well with dementia: a National Dementia Strategy. London: Department of Health; 2009.

[26] Warren S, Fey M, Yoder P. Differential treatment intensity research: a missing link to creating optimally effective communication interventions. Ment Retard Dev Disabil Res Rev. 2007;13:70–7. 17326112 10.1002/mrdd.20139

[27] Edemekong P, Bomgaars D, Sukumaran S, Schoo C. Activities of Daily Living. 2023 Jun 26. StatPearls [Internet]. Treasure Island (FL): StatPearls Publishing; 2024 Jan–. PMID: 29261878. 29261878

[28] Akintola A, Achterberg W, Caljouw M. Non-pharmacological interventions for improving quality of life of long-term care residents with dementia: a scoping review protocol. BMJ Open. 2019;9(12):e032661. 31874881 10.1136/bmjopen-2019-032661 PMC7008431

[29] Berg-Weger M, Stewart D. Non-pharmacologic interventions for persons with dementia. Mo Med. 2017;114(2):116–19. 30228557 PMC6140014

[30] Veld HIH, van Asch J, Willemse I, Verkade B, Pot P, Blom A, Groot Zwaaftink M, Francke R. Process evaluation of nurse-led online self-management support for family caregivers to deal with behaviour changes of a relative with dementia (part 1): mixed methods study. J Med Internet Res. 2019;21(10):e13002. 31605517 10.2196/13002 PMC6914225

[31] Veld HIH, Willemse J, van Asch B, Groot Zwaaftink I, Verkade R, Twisk P, Verkaik J, Blom R, van Meijel M, Francke B. Online self-management support for family caregivers dealing with behaviour changes in relatives with dementia (part 2): randomized controlled trial. J Med Internet Res. 2020;22(2):e13001. 32130142 10.2196/13001 PMC7064946

[32] Mays N, Roberts E, Popay J. Synthesising research evidence. In: Fulop N, Allen P, Clarke A, Black N, editors. Studying the Organization and Delivery of Health Services: Research methods. London: Routledge; 2001. pp. 188–220.

[33] Aromataris E, Munn Z. Joanna Briggs Institute Manual for Evidence Synthesis; 2020. https://jbi-global-wiki.refined.site/space/MANUAL. Accessed 18 July 2024.

[34] Skivington K, Matthews L, Simpson S, Craig P, Baird J, Blazeby J, Boyd K, Craig N, French D, McIntosh E, Petticrew M, Rycroft-Malone J, White M, Moore L. A new framework for developing and evaluating complex interventions: update of Medical Research Council guidance. BMJ. 2021; 374: n2061. 10.1136/bmj.n2061 PMC8482308 34593508

[35] Hong Q, Pluye P, Fàbregues S, Bartlett G, Boardman F, Cargo M, Dagenais P, Gagnon M-P, Griffiths F, Nicolau B, O’Cathain A, Rousseau M-C, Vedel I. Mixed-methods Appraisal Tool (MMAT) Version 2018. User Guide; 2018. 10.1016/j.jclinepi.2019.03.008 30905698

[36] Mays N, Pope C, Popay J. Systematically reviewing qualitative and quantitative evidence to inform management and policy-making in the health field. J Health Serv Res Policy. 2005;10(1):6–20. 16053580 10.1258/1355819054308576

[37] Popay J, Roberts H, Sowden A, Petticrew M, Arai L, Rodgers M, Britten N, Roen K, Duffy S. Guidance on the conduct of narrative synthesis in systematic reviews: a product from the ESRC methods Programme. 2006; 10.13140/2.1.1018.4643

[38] van der Roest H, Meiland F, Jonker C, Dröes R-M. User evaluation of the DEMentia-specific Digital Interactive Social Chart (DEM-DISC). A pilot study among informal carers on its impact, user friendliness and usefulness. Aging Ment Health. 2010;14(4):461–70. 20455122 10.1080/13607860903311741

[39] Van Mierlo L, Meiland F, Van de Ven P, Van Hout H, Dröes R. Evaluation of DEM-DISC, customized e-advice on health and social support services for informal carers and case managers of people with dementia; a cluster randomized trial. Int Psychogeriatr. 2015;27(8):1365–78. 25872457 10.1017/S1041610215000423

[40] Barry H, Bedford L, McGrattan M, Ryan C, Passmore P, Robinson L, Molloy G, Darcy C, Buchanan H, Hughes C. Improving medicines management for people with dementia in primary care: a qualitative study of healthcare professionals to develop a theory-informed intervention. BMC Health Serv Res. 2020;20:120. 32059718 10.1186/s12913-020-4971-7 PMC7023803

[41] Cristancho-Lacroix V, Wrobel J, Cantegreil-Kallen I, Dub T, Rouquette A, Rigaud A. A web-based psychoeducational program for informal caregivers of patients with Alzheimer’s disease: a pilot randomized controlled trial. J Med Internet Res. 2015;17(5):e117. 25967983 10.2196/jmir.3717 PMC4468784

[42] Gaugler J, Hobday J, Robbins J, Barclay M. CARES(^®^) dementia care for Families™: effects of Online, Psychoeducational training on knowledge of person-centered care and satisfaction. J Gerontol Nurs. 2015;41(10):18–24. 26270065 10.3928/00989134-20150804-61 PMC5769472

[43] Gies C, Pierce L. The Journey of web-based education for caregivers of persons with dementia: it didn’t happen overnight. Home Healthc Now. 2021;39(3):160–8. 33955930 10.1097/NHH.0000000000000960

[44] Lewis M, Hobday J, Hepburn K. Internet-based program for Dementia caregivers. Am J Alzheimers Dis Other. 2010;25(8):674–9. 10.1177/1533317510385812 PMC10845646 21131674

[45] Metcalfe A, Jones B, Mayer J, Gage H, Oyebode J, Boucault S, Aloui S, Schwertel U, Böhm M, Tezenas du Montcel S, Lebbah S, De Mendonça A, De Vugt M, Graff C, Jansen S, Hergueta T, Dubois B, Kurz A. Online information and support for carers of people with young-onset dementia: a multi-site randomised controlled pilot study. Int J Geriatr Psychiatry. 2019;34(10):1455–64. 31111516 10.1002/gps.5154

[46] Meyer C, Dow B, Hill K, Tinney J, Hill S. The right way at the right time: insights on the Uptake of Falls Prevention Strategies from people with Dementia and their caregivers. Front Public Health. 2016;4. 10.3389/fpubh.2016.00244. 10.3389/fpubh.2016.00244 PMC5090973 27853730

[47] Yates L, Csipke E, Moniz-Cook E, Leung P, Walton H, Charlesworth G, Spector A, Hogervorst E, Mountain G, Orrell M. The development of the promoting independence in dementia (PRIDE) intervention to enhance independence in dementia. Clin Interv Aging. 2019;14:1615–30. 31571842 10.2147/CIA.S214367 PMC6748161

[48] Sbaffi L, Hargreaves S. The information trust formation process for informal caregivers of people with dementia: a qualitative study. J Doc. 2022;78(2):302–19. 10.1108/JD-01-2021-0014.

[49] Rowley J, Johnson F, Sbaffi L. Gender as an influencer of online health information seeking and evaluation behaviour. J Assoc Inf Sci Technol. 2017;68(1):36–47.

[50] Erdelez S, Tanacković S, Balog K. Online behaviour of the Alzheimer’s disease patient caregivers on Croatian online discussion forum. Proc Association Inform Sci Technol. 2019;56(1):78–88.

[51] Balog K, Tanacković S, Erdelez S. Information support system for Alzheimer’s disease patients’ caregivers in Croatia: a phenomenological approach. Inform Res. 2020;25(4). 10.47989/irisic2011.

[52] Peterson K, Hahn H, Lee A, Madison C, Atri A. In the Information Age, do dementia caregivers get the information they need? Semi-structured interviews to determine informal caregivers’ education needs, barriers, and preferences. BMC Geriatr. 2016;16(1):164. 10.1186/s12877-016-0338-7. 27662829 10.1186/s12877-016-0338-7 PMC5035467

[53] Maneze D, Weaver R, Kovai V, Salamonson Y, Astorga C, Yogendran D, Everett B. Some say no, some say yes: receiving inconsistent or insufficient information from healthcare professionals and consequences for diabetes self-management: a qualitative study in patients with type 2 diabetes. Diabetes Res Clin Pract. 2019;156:107830. 10.1016/j.diabres.2019.107830. 31465812 10.1016/j.diabres.2019.107830

[54] Litzkendorf S, Frank M, Babac A, Rosenfeldt D, Schauer F, Hartz T, von der Graf JM. Use and importance of different information sources among patients with rare diseases and their relatives over time: a qualitative study. BMC Public Health. 2020;20:860. 32503483 10.1186/s12889-020-08926-9 PMC7275578

[55] Harrop E, Byrne A, Nelson A. It’s alright to ask for help: findings from a qualitative study exploring the information and support needs of family carers at the end of life. BMC Palliat Care. 2014;13:22. 24742046 10.1186/1472-684X-13-22 PMC3997794

[56] Hassan A, Bronzini M, Lamura G. Digital technologies as sources of information for patients and caregivers during COVID-19 pandemic: a cross-sectional survey. Digit Health. 2023;9. 10.1177/20552076231156214. 10.1177/20552076231156214 PMC9996721 36908378

[57] Hoffmann T, Glasziou P, Boutron I, Milne R, Perera R, Moher D, Altman D, Barbour V, Macdonald H, Johnston M, Lamb S, Dixon-Woods M, McCulloch P, Wyatt J, Chan A-W, Michie S. Better reporting of interventions: template for intervention description and replication (TIDieR) checklist and guide. BMJ. 2014;348:g1687. 10.1136/bmj.g1687. 24609605 10.1136/bmj.g1687

