# Supplementary material for: Interventions delivered by primary or community healthcare professionals to support people living at home with dementia with activities of daily living: a systematic review and narrative synthesis
Source: BMC Geriatr. 2024 Oct 23;24:860. doi: 10.1186/s12877-024-05465-5 (PMC11515696; doi:10.1186/s12877-024-05465-5)
Supplement: Supplementary file 1 — Supplementary Material 1 [file 12877_2024_5465_MOESM1_ESM.docx]

**Additional File 1 Search terms for each database**

**Table 1: OVID MEDLINE**

| 1 | non-pharmacologic*.mp. [mp=title, book title, abstract, original title, name of substance word, subject heading word, floating sub-heading word, keyword heading word, organism supplementary concept word, protocol supplementary concept word, rare disease supplementary concept word, unique identifier, synonyms] |
| --- | --- |
| 2 | nonpharmacologic*.mp. [mp=title, book title, abstract, original title, name of substance word, subject heading word, floating sub-heading word, keyword heading word, organism supplementary concept word, protocol supplementary concept word, rare disease supplementary concept word, unique identifier, synonyms] |
| 3 | behavio* therap*.mp. [mp=title, book title, abstract, original title, name of substance word, subject heading word, floating sub-heading word, keyword heading word, organism supplementary concept word, protocol supplementary concept word, rare disease supplementary concept word, unique identifier, synonyms] |
| 4 | exp Behavior Therapy/ |
| 5 | intervention*.mp. [mp=title, book title, abstract, original title, name of substance word, subject heading word, floating sub-heading word, keyword heading word, organism supplementary concept word, protocol supplementary concept word, rare disease supplementary concept word, unique identifier, synonyms] |
| 6 | exp Early Intervention, Educational/ or exp Psychosocial Intervention/ or exp Internet-Based Intervention/ |
| 7 | innovation*.mp. [mp=title, book title, abstract, original title, name of substance word, subject heading word, floating sub-heading word, keyword heading word, organism supplementary concept word, protocol supplementary concept word, rare disease supplementary concept word, unique identifier, synonyms] |
| 8 | initiative*.mp. [mp=title, book title, abstract, original title, name of substance word, subject heading word, floating sub-heading word, keyword heading word, organism supplementary concept word, protocol supplementary concept word, rare disease supplementary concept word, unique identifier, synonyms] |
| 9 | psycho-social.mp. [mp=title, book title, abstract, original title, name of substance word, subject heading word, floating sub-heading word, keyword heading word, organism supplementary concept word, protocol supplementary concept word, rare disease supplementary concept word, unique identifier, synonyms] |
| 10 | psychosocial.mp. [mp=title, book title, abstract, original title, name of substance word, subject heading word, floating sub-heading word, keyword heading word, organism supplementary concept word, protocol supplementary concept word, rare disease supplementary concept word, unique identifier, synonyms] |
| 11 | exp Psychosocial Intervention/ |
| 12 | nondrug.mp. [mp=title, book title, abstract, original title, name of substance word, subject heading word, floating sub-heading word, keyword heading word, organism supplementary concept word, protocol supplementary concept word, rare disease supplementary concept word, unique identifier, synonyms] |
| 13 | non-drug.mp. [mp=title, book title, abstract, original title, name of substance word, subject heading word, floating sub-heading word, keyword heading word, organism supplementary concept word, protocol supplementary concept word, rare disease supplementary concept word, unique identifier, synonyms] |
| 14 | general practi*.mp. [mp=title, book title, abstract, original title, name of substance word, subject heading word, floating sub-heading word, keyword heading word, organism supplementary concept word, protocol supplementary concept word, rare disease supplementary concept word, unique identifier, synonyms] |
| 15 | exp General Practice/ or exp Family Practice/ |
| 16 | exp Community Health Centers/ |
| 17 | exp General Practitioners/ or exp Physicians, Family/ |
| 18 | physician*.mp. [mp=title, book title, abstract, original title, name of substance word, subject heading word, floating sub-heading word, keyword heading word, organism supplementary concept word, protocol supplementary concept word, rare disease supplementary concept word, unique identifier, synonyms] |
| 19 | exp Physicians/ |
| 20 | exp Geriatricians/ |
| 21 | geriatrician*.mp. [mp=title, book title, abstract, original title, name of substance word, subject heading word, floating sub-heading word, keyword heading word, organism supplementary concept word, protocol supplementary concept word, rare disease supplementary concept word, unique identifier, synonyms] |
| 22 | nurse*.mp. [mp=title, book title, abstract, original title, name of substance word, subject heading word, floating sub-heading word, keyword heading word, organism supplementary concept word, protocol supplementary concept word, rare disease supplementary concept word, unique identifier, synonyms] |
| 23 | nursing.mp. [mp=title, book title, abstract, original title, name of substance word, subject heading word, floating sub-heading word, keyword heading word, organism supplementary concept word, protocol supplementary concept word, rare disease supplementary concept word, unique identifier, synonyms] |
| 24 | exp Community Health Nursing/ or exp Geriatric Nursing/ or exp Home Health Nursing/ or exp Home Nursing/ or exp Nursing/ |
| 25 | exp Nurses, Community Health/ or exp Nurses/ or exp Nurses Improving Care for Health System Elders/ |
| 26 | occupational therap*.mp. [mp=title, book title, abstract, original title, name of substance word, subject heading word, floating sub-heading word, keyword heading word, organism supplementary concept word, protocol supplementary concept word, rare disease supplementary concept word, unique identifier, synonyms] |
| 27 | exp Occupational Therapy/ or exp Occupational Therapists/ |
| 28 | physiotherap*.mp. [mp=title, book title, abstract, original title, name of substance word, subject heading word, floating sub-heading word, keyword heading word, organism supplementary concept word, protocol supplementary concept word, rare disease supplementary concept word, unique identifier, synonyms] |
| 29 | physical therap*.mp. [mp=title, book title, abstract, original title, name of substance word, subject heading word, floating sub-heading word, keyword heading word, organism supplementary concept word, protocol supplementary concept word, rare disease supplementary concept word, unique identifier, synonyms] |
| 30 | exp Physical Therapists/ or exp Physical Therapy Modalities/ or exp Physical Therapy Specialty/ |
| 31 | health care profession*.mp. [mp=title, book title, abstract, original title, name of substance word, subject heading word, floating sub-heading word, keyword heading word, organism supplementary concept word, protocol supplementary concept word, rare disease supplementary concept word, unique identifier, synonyms] |
| 32 | exp Health Personnel/ |
| 33 | healthcare profession*.mp. [mp=title, book title, abstract, original title, name of substance word, subject heading word, floating sub-heading word, keyword heading word, organism supplementary concept word, protocol supplementary concept word, rare disease supplementary concept word, unique identifier, synonyms] |
| 34 | primary healthcare.mp. [mp=title, book title, abstract, original title, name of substance word, subject heading word, floating sub-heading word, keyword heading word, organism supplementary concept word, protocol supplementary concept word, rare disease supplementary concept word, unique identifier, synonyms] |
| 35 | primary health care.mp. [mp=title, book title, abstract, original title, name of substance word, subject heading word, floating sub-heading word, keyword heading word, organism supplementary concept word, protocol supplementary concept word, rare disease supplementary concept word, unique identifier, synonyms] |
| 36 | exp Primary Health Care/ |
| 37 | community health.mp. [mp=title, book title, abstract, original title, name of substance word, subject heading word, floating sub-heading word, keyword heading word, organism supplementary concept word, protocol supplementary concept word, rare disease supplementary concept word, unique identifier, synonyms] |
| 38 | primary care.mp. [mp=title, book title, abstract, original title, name of substance word, subject heading word, floating sub-heading word, keyword heading word, organism supplementary concept word, protocol supplementary concept word, rare disease supplementary concept word, unique identifier, synonyms] |
| 39 | GP.mp. [mp=title, book title, abstract, original title, name of substance word, subject heading word, floating sub-heading word, keyword heading word, organism supplementary concept word, protocol supplementary concept word, rare disease supplementary concept word, unique identifier, synonyms] |
| 40 | dement*.mp. [mp=title, book title, abstract, original title, name of substance word, subject heading word, floating sub-heading word, keyword heading word, organism supplementary concept word, protocol supplementary concept word, rare disease supplementary concept word, unique identifier, synonyms] |
| 41 | exp Dementia, Vascular/ or exp Dementia/ or exp Frontotemporal Dementia/ or exp Dementia, Multi-Infarct/ |
| 42 | alzheimer*.mp. [mp=title, book title, abstract, original title, name of substance word, subject heading word, floating sub-heading word, keyword heading word, organism supplementary concept word, protocol supplementary concept word, rare disease supplementary concept word, unique identifier, synonyms] |
| 43 | exp Alzheimer Disease/ |
| 44 | community care.mp. [mp=title, book title, abstract, original title, name of substance word, subject heading word, floating sub-heading word, keyword heading word, organism supplementary concept word, protocol supplementary concept word, rare disease supplementary concept word, unique identifier, synonyms] |
| 45 | exp Community Health Services/ |
| 46 | independent living.mp. [mp=title, book title, abstract, original title, name of substance word, subject heading word, floating sub-heading word, keyword heading word, organism supplementary concept word, protocol supplementary concept word, rare disease supplementary concept word, unique identifier, synonyms] |
| 47 | exp Independent Living/ |
| 48 | community dwelling*.mp. [mp=title, book title, abstract, original title, name of substance word, subject heading word, floating sub-heading word, keyword heading word, organism supplementary concept word, protocol supplementary concept word, rare disease supplementary concept word, unique identifier, synonyms] |
| 49 | community-dwelling*.mp. [mp=title, book title, abstract, original title, name of substance word, subject heading word, floating sub-heading word, keyword heading word, organism supplementary concept word, protocol supplementary concept word, rare disease supplementary concept word, unique identifier, synonyms] |
| 50 | community living.mp. [mp=title, book title, abstract, original title, name of substance word, subject heading word, floating sub-heading word, keyword heading word, organism supplementary concept word, protocol supplementary concept word, rare disease supplementary concept word, unique identifier, synonyms] |
| 51 | community-living.mp. [mp=title, book title, abstract, original title, name of substance word, subject heading word, floating sub-heading word, keyword heading word, organism supplementary concept word, protocol supplementary concept word, rare disease supplementary concept word, unique identifier, synonyms] |
| 52 | home-based.mp. [mp=title, book title, abstract, original title, name of substance word, subject heading word, floating sub-heading word, keyword heading word, organism supplementary concept word, protocol supplementary concept word, rare disease supplementary concept word, unique identifier, synonyms] |
| 53 | home based.mp. [mp=title, book title, abstract, original title, name of substance word, subject heading word, floating sub-heading word, keyword heading word, organism supplementary concept word, protocol supplementary concept word, rare disease supplementary concept word, unique identifier, synonyms] |
| 54 | (liv* adj6 house).mp. [mp=title, book title, abstract, original title, name of substance word, subject heading word, floating sub-heading word, keyword heading word, organism supplementary concept word, protocol supplementary concept word, rare disease supplementary concept word, unique identifier, synonyms] |
| 55 | (resid* adj6 house).mp. [mp=title, book title, abstract, original title, name of substance word, subject heading word, floating sub-heading word, keyword heading word, organism supplementary concept word, protocol supplementary concept word, rare disease supplementary concept word, unique identifier, synonyms] |
| 56 | (liv* adj6 home).mp. [mp=title, book title, abstract, original title, name of substance word, subject heading word, floating sub-heading word, keyword heading word, organism supplementary concept word, protocol supplementary concept word, rare disease supplementary concept word, unique identifier, synonyms] |
| 57 | (resid* adj6 home).mp. [mp=title, book title, abstract, original title, name of substance word, subject heading word, floating sub-heading word, keyword heading word, organism supplementary concept word, protocol supplementary concept word, rare disease supplementary concept word, unique identifier, synonyms] |
| 58 | (liv* adj6 community).mp. [mp=title, book title, abstract, original title, name of substance word, subject heading word, floating sub-heading word, keyword heading word, organism supplementary concept word, protocol supplementary concept word, rare disease supplementary concept word, unique identifier, synonyms] |
| 59 | (resid* adj6 community).mp. [mp=title, book title, abstract, original title, name of substance word, subject heading word, floating sub-heading word, keyword heading word, organism supplementary concept word, protocol supplementary concept word, rare disease supplementary concept word, unique identifier, synonyms] |
| 60 | house bound.mp. [mp=title, book title, abstract, original title, name of substance word, subject heading word, floating sub-heading word, keyword heading word, organism supplementary concept word, protocol supplementary concept word, rare disease supplementary concept word, unique identifier, synonyms] |
| 61 | housebound.mp. [mp=title, book title, abstract, original title, name of substance word, subject heading word, floating sub-heading word, keyword heading word, organism supplementary concept word, protocol supplementary concept word, rare disease supplementary concept word, unique identifier, synonyms] |
| 62 | homebound.mp. [mp=title, book title, abstract, original title, name of substance word, subject heading word, floating sub-heading word, keyword heading word, organism supplementary concept word, protocol supplementary concept word, rare disease supplementary concept word, unique identifier, synonyms] |
| 63 | home bound.mp. [mp=title, book title, abstract, original title, name of substance word, subject heading word, floating sub-heading word, keyword heading word, organism supplementary concept word, protocol supplementary concept word, rare disease supplementary concept word, unique identifier, synonyms] |
| 64 | exp Homebound Persons/ |
| 65 | 1 or 2 or 3 or 4 or 5 or 6 or 7 or 8 or 9 or 10 or 11 or 12 or 13 |
| 66 | 14 or 15 or 16 or 17 or 18 or 19 or 20 or 21 or 22 or 23 or 24 or 25 or 26 or 27 or 28 or 29 or 30 or 31 or 32 or 33 or 34 or 35 or 36 or 37 or 38 or 39 or 44 or 45 |
| 67 | 40 or 41 or 42 or 43 |
| 68 | 46 or 47 or 48 or 49 or 50 or 51 or 52 or 53 or 54 or 55 or 56 or 57 or 58 or 59 or 60 or 61 or 62 or 63 or 64 |
| 69 | 65 and 66 and 67 and 68 |
| 70 | limit 69 to (english language and yr="2009 - 2022") |

**Table 2: Ovid PsycINFO**

| 1 | non-pharmacologic*.mp. [mp=title, abstract, heading word, table of contents, key concepts, original title, tests & measures, mesh word] |
| --- | --- |
| 2 | nonpharmacologic*.mp. [mp=title, abstract, heading word, table of contents, key concepts, original title, tests & measures, mesh word] |
| 3 | behavio* therap*.mp. [mp=title, abstract, heading word, table of contents, key concepts, original title, tests & measures, mesh word] |
| 4 | exp Behavior Therapy/ |
| 5 | intervention*.mp. [mp=title, abstract, heading word, table of contents, key concepts, original title, tests & measures, mesh word] |
| 6 | exp Early Intervention, Educational/ or exp Psychosocial Intervention/ or exp Internet-Based Intervention/ |
| 7 | innovation*.mp. [mp=title, abstract, heading word, table of contents, key concepts, original title, tests & measures, mesh word] |
| 8 | initiative*.mp. [mp=title, abstract, heading word, table of contents, key concepts, original title, tests & measures, mesh word] |
| 9 | psycho-social.mp. [mp=title, abstract, heading word, table of contents, key concepts, original title, tests & measures, mesh word] |
| 10 | psychosocial.mp. [mp=title, abstract, heading word, table of contents, key concepts, original title, tests & measures, mesh word] |
| 11 | exp Psychosocial Intervention/ |
| 12 | nondrug.mp. [mp=title, abstract, heading word, table of contents, key concepts, original title, tests & measures, mesh word] |
| 13 | non-drug.mp. [mp=title, abstract, heading word, table of contents, key concepts, original title, tests & measures, mesh word] |
| 14 | general practi*.mp. [mp=title, abstract, heading word, table of contents, key concepts, original title, tests & measures, mesh word] |
| 15 | exp General Practice/ or exp Family Practice/ |
| 16 | exp Community Health Centers/ |
| 17 | exp General Practitioners/ or exp Physicians, Family/ |
| 18 | physician*.mp. [mp=title, abstract, heading word, table of contents, key concepts, original title, tests & measures, mesh word] |
| 19 | exp Physicians/ |
| 20 | exp Geriatricians/ |
| 21 | geriatrician*.mp. [mp=title, abstract, heading word, table of contents, key concepts, original title, tests & measures, mesh word] |
| 22 | nurse*.mp. [mp=title, abstract, heading word, table of contents, key concepts, original title, tests & measures, mesh word] |
| 23 | nursing.mp. [mp=title, abstract, heading word, table of contents, key concepts, original title, tests & measures, mesh word] |
| 24 | exp Community Health Nursing/ or exp Geriatric Nursing/ or exp Home Health Nursing/ or exp Home Nursing/ or exp Nursing/ |
| 25 | exp Nurses, Community Health/ or exp Nurses/ or exp Nurses Improving Care for Health System Elders/ |
| 26 | occupational therap*.mp. [mp=title, abstract, heading word, table of contents, key concepts, original title, tests & measures, mesh word] |
| 27 | exp Occupational Therapy/ or exp Occupational Therapists/ |
| 28 | physiotherap*.mp. [mp=title, abstract, heading word, table of contents, key concepts, original title, tests & measures, mesh word] |
| 29 | physical therap*.mp. [mp=title, abstract, heading word, table of contents, key concepts, original title, tests & measures, mesh word] |
| 30 | exp Physical Therapists/ or exp Physical Therapy Modalities/ or exp Physical Therapy Specialty/ |
| 31 | health care profession*.mp. [mp=title, abstract, heading word, table of contents, key concepts, original title, tests & measures, mesh word] |
| 32 | exp Health Personnel/ |
| 33 | healthcare profession*.mp. [mp=title, abstract, heading word, table of contents, key concepts, original title, tests & measures, mesh word] |
| 34 | primary healthcare.mp. [mp=title, abstract, heading word, table of contents, key concepts, original title, tests & measures, mesh word] |
| 35 | primary health care.mp. [mp=title, abstract, heading word, table of contents, key concepts, original title, tests & measures, mesh word] |
| 36 | exp Primary Health Care/ |
| 37 | community health.mp. [mp=title, abstract, heading word, table of contents, key concepts, original title, tests & measures, mesh word] |
| 38 | primary care.mp. [mp=title, abstract, heading word, table of contents, key concepts, original title, tests & measures, mesh word] |
| 39 | GP.mp. [mp=title, abstract, heading word, table of contents, key concepts, original title, tests & measures, mesh word] |
| 40 | dement*.mp. [mp=title, abstract, heading word, table of contents, key concepts, original title, tests & measures, mesh word] |
| 41 | exp Dementia, Vascular/ or exp Dementia/ or exp Frontotemporal Dementia/ or exp Dementia, Multi-Infarct/ |
| 42 | alzheimer*.mp. [mp=title, abstract, heading word, table of contents, key concepts, original title, tests & measures, mesh word] |
| 43 | exp Alzheimer Disease/ |
| 44 | community care.mp. [mp=title, abstract, heading word, table of contents, key concepts, original title, tests & measures, mesh word] |
| 45 | exp Community Health Services/ |
| 46 | independent living.mp. [mp=title, abstract, heading word, table of contents, key concepts, original title, tests & measures, mesh word] |
| 47 | exp Independent Living/ |
| 48 | community dwelling*.mp. [mp=title, abstract, heading word, table of contents, key concepts, original title, tests & measures, mesh word] |
| 49 | community-dwelling*.mp. [mp=title, abstract, heading word, table of contents, key concepts, original title, tests & measures, mesh word] |
| 50 | community living.mp. [mp=title, abstract, heading word, table of contents, key concepts, original title, tests & measures, mesh word] |
| 51 | community-living.mp. [mp=title, abstract, heading word, table of contents, key concepts, original title, tests & measures, mesh word] |
| 52 | home-based.mp. [mp=title, abstract, heading word, table of contents, key concepts, original title, tests & measures, mesh word] |
| 53 | home based.mp. [mp=title, abstract, heading word, table of contents, key concepts, original title, tests & measures, mesh word] |
| 54 | (liv* adj6 house).mp. [mp=title, abstract, heading word, table of contents, key concepts, original title, tests & measures, mesh word] |
| 55 | (resid* adj6 house).mp. [mp=title, abstract, heading word, table of contents, key concepts, original title, tests & measures, mesh word] |
| 56 | (liv* adj6 home).mp. [mp=title, abstract, heading word, table of contents, key concepts, original title, tests & measures, mesh word] |
| 57 | (resid* adj6 home).mp. [mp=title, abstract, heading word, table of contents, key concepts, original title, tests & measures, mesh word] |
| 58 | (liv* adj6 community).mp. [mp=title, abstract, heading word, table of contents, key concepts, original title, tests & measures, mesh word] |
| 59 | (resid* adj6 community).mp. [mp=title, abstract, heading word, table of contents, key concepts, original title, tests & measures, mesh word] |
| 60 | house bound.mp. [mp=title, abstract, heading word, table of contents, key concepts, original title, tests & measures, mesh word] |
| 61 | housebound.mp. [mp=title, abstract, heading word, table of contents, key concepts, original title, tests & measures, mesh word] |
| 62 | homebound.mp. [mp=title, abstract, heading word, table of contents, key concepts, original title, tests & measures, mesh word] |
| 63 | home bound.mp. [mp=title, abstract, heading word, table of contents, key concepts, original title, tests & measures, mesh word] |
| 64 | exp Homebound Persons/ |
| 65 | 1 or 2 or 3 or 4 or 5 or 6 or 7 or 8 or 9 or 10 or 11 or 12 or 13 |
| 66 | 14 or 15 or 16 or 17 or 18 or 19 or 20 or 21 or 22 or 23 or 24 or 25 or 26 or 27 or 28 or 29 or 30 or 31 or 32 or 33 or 34 or 35 or 36 or 37 or 38 or 39 or 44 or 45 |
| 67 | 40 or 41 or 42 or 43 |
| 68 | 46 or 47 or 48 or 49 or 50 or 51 or 52 or 53 or 54 or 55 or 56 or 57 or 58 or 59 or 60 or 61 or 62 or 63 or 64 |
| 69 | 65 and 66 and 67 and 68 |
| 70 | limit 69 to (english language and yr="2009 - 2022") |
| 71 | exp Psychosocial Rehabilitation/ |
| 72 | exp Geriatrics/ or exp Geriatric Patients/ or exp Gerontology/ |
| 73 | exp Community Health/ or exp General Practitioners/ or exp Community Services/ |
| 74 | exp Primary Health Care/ |
| 75 | exp Homebound/ |
| 76 | exp Response to Intervention/ or exp Early Intervention/ or exp Intervention/ |
| 77 | exp Family Physicians/ |
| 78 | 65 or 71 or 76 |
| 79 | 66 or 72 or 73 or 74 or 77 |
| 80 | 68 or 75 |
| 81 | 67 and 78 and 79 and 80 |
| 82 | limit 81 to (english language and yr="2009 - 2022") |

**Table 3: Embase**

| 1 | non-pharmacologic*.mp. [mp=title, abstract, heading word, drug trade name, original title, device manufacturer, drug manufacturer, device trade name, keyword heading word, floating subheading word, candidate term word] |
| --- | --- |
| 2 | nonpharmacologic*.mp. [mp=title, abstract, heading word, drug trade name, original title, device manufacturer, drug manufacturer, device trade name, keyword heading word, floating subheading word, candidate term word] |
| 3 | behavio* therap*.mp. [mp=title, abstract, heading word, drug trade name, original title, device manufacturer, drug manufacturer, device trade name, keyword heading word, floating subheading word, candidate term word] |
| 4 | exp Behavior Therapy/ |
| 5 | intervention*.mp. [mp=title, abstract, heading word, drug trade name, original title, device manufacturer, drug manufacturer, device trade name, keyword heading word, floating subheading word, candidate term word] |
| 6 | exp Early Intervention, Educational/ or exp Psychosocial Intervention/ or exp Internet-Based Intervention/ |
| 7 | innovation*.mp. [mp=title, abstract, heading word, drug trade name, original title, device manufacturer, drug manufacturer, device trade name, keyword heading word, floating subheading word, candidate term word] |
| 8 | initiative*.mp. [mp=title, abstract, heading word, drug trade name, original title, device manufacturer, drug manufacturer, device trade name, keyword heading word, floating subheading word, candidate term word] |
| 9 | psycho-social.mp. [mp=title, abstract, heading word, drug trade name, original title, device manufacturer, drug manufacturer, device trade name, keyword heading word, floating subheading word, candidate term word] |
| 10 | psychosocial.mp. [mp=title, abstract, heading word, drug trade name, original title, device manufacturer, drug manufacturer, device trade name, keyword heading word, floating subheading word, candidate term word] |
| 11 | exp Psychosocial Intervention/ |
| 12 | nondrug.mp. [mp=title, abstract, heading word, drug trade name, original title, device manufacturer, drug manufacturer, device trade name, keyword heading word, floating subheading word, candidate term word] |
| 13 | non-drug.mp. [mp=title, abstract, heading word, drug trade name, original title, device manufacturer, drug manufacturer, device trade name, keyword heading word, floating subheading word, candidate term word] |
| 14 | general practi*.mp. [mp=title, abstract, heading word, drug trade name, original title, device manufacturer, drug manufacturer, device trade name, keyword heading word, floating subheading word, candidate term word] |
| 15 | exp General Practice/ or exp Family Practice/ |
| 16 | exp Community Health Centers/ |
| 17 | exp General Practitioners/ or exp Physicians, Family/ |
| 18 | physician*.mp. [mp=title, abstract, heading word, drug trade name, original title, device manufacturer, drug manufacturer, device trade name, keyword heading word, floating subheading word, candidate term word] |
| 19 | exp Physicians/ |
| 20 | exp Geriatricians/ |
| 21 | geriatrician*.mp. [mp=title, abstract, heading word, drug trade name, original title, device manufacturer, drug manufacturer, device trade name, keyword heading word, floating subheading word, candidate term word] |
| 22 | nurse*.mp. [mp=title, abstract, heading word, drug trade name, original title, device manufacturer, drug manufacturer, device trade name, keyword heading word, floating subheading word, candidate term word] |
| 23 | nursing.mp. [mp=title, abstract, heading word, drug trade name, original title, device manufacturer, drug manufacturer, device trade name, keyword heading word, floating subheading word, candidate term word] |
| 24 | exp Community Health Nursing/ or exp Geriatric Nursing/ or exp Home Health Nursing/ or exp Home Nursing/ or exp Nursing/ |
| 25 | exp Nurses, Community Health/ or exp Nurses/ or exp Nurses Improving Care for Health System Elders/ |
| 26 | occupational therap*.mp. [mp=title, abstract, heading word, drug trade name, original title, device manufacturer, drug manufacturer, device trade name, keyword heading word, floating subheading word, candidate term word] |
| 27 | exp Occupational Therapy/ or exp Occupational Therapists/ |
| 28 | physiotherap*.mp. [mp=title, abstract, heading word, drug trade name, original title, device manufacturer, drug manufacturer, device trade name, keyword heading word, floating subheading word, candidate term word] |
| 29 | physical therap*.mp. [mp=title, abstract, heading word, drug trade name, original title, device manufacturer, drug manufacturer, device trade name, keyword heading word, floating subheading word, candidate term word] |
| 30 | exp Physical Therapists/ or exp Physical Therapy Modalities/ or exp Physical Therapy Specialty/ |
| 31 | health care profession*.mp. [mp=title, abstract, heading word, drug trade name, original title, device manufacturer, drug manufacturer, device trade name, keyword heading word, floating subheading word, candidate term word] |
| 32 | exp Health Personnel/ |
| 33 | healthcare profession*.mp. [mp=title, abstract, heading word, drug trade name, original title, device manufacturer, drug manufacturer, device trade name, keyword heading word, floating subheading word, candidate term word] |
| 34 | primary healthcare.mp. [mp=title, abstract, heading word, drug trade name, original title, device manufacturer, drug manufacturer, device trade name, keyword heading word, floating subheading word, candidate term word] |
| 35 | primary health care.mp. [mp=title, abstract, heading word, drug trade name, original title, device manufacturer, drug manufacturer, device trade name, keyword heading word, floating subheading word, candidate term word] |
| 36 | exp Primary Health Care/ |
| 37 | community health.mp. [mp=title, abstract, heading word, drug trade name, original title, device manufacturer, drug manufacturer, device trade name, keyword heading word, floating subheading word, candidate term word] |
| 38 | primary care.mp. [mp=title, abstract, heading word, drug trade name, original title, device manufacturer, drug manufacturer, device trade name, keyword heading word, floating subheading word, candidate term word] |
| 39 | GP.mp. [mp=title, abstract, heading word, drug trade name, original title, device manufacturer, drug manufacturer, device trade name, keyword heading word, floating subheading word, candidate term word] |
| 40 | dement*.mp. [mp=title, abstract, heading word, drug trade name, original title, device manufacturer, drug manufacturer, device trade name, keyword heading word, floating subheading word, candidate term word] |
| 41 | exp Dementia, Vascular/ or exp Dementia/ or exp Frontotemporal Dementia/ or exp Dementia, Multi-Infarct/ |
| 42 | alzheimer*.mp. [mp=title, abstract, heading word, drug trade name, original title, device manufacturer, drug manufacturer, device trade name, keyword heading word, floating subheading word, candidate term word] |
| 43 | exp Alzheimer Disease/ |
| 44 | community care.mp. [mp=title, abstract, heading word, drug trade name, original title, device manufacturer, drug manufacturer, device trade name, keyword heading word, floating subheading word, candidate term word] |
| 45 | exp Community Health Services/ |
| 46 | independent living.mp. [mp=title, abstract, heading word, drug trade name, original title, device manufacturer, drug manufacturer, device trade name, keyword heading word, floating subheading word, candidate term word] |
| 47 | exp Independent Living/ |
| 48 | community dwelling*.mp. [mp=title, abstract, heading word, drug trade name, original title, device manufacturer, drug manufacturer, device trade name, keyword heading word, floating subheading word, candidate term word] |
| 49 | community-dwelling*.mp. [mp=title, abstract, heading word, drug trade name, original title, device manufacturer, drug manufacturer, device trade name, keyword heading word, floating subheading word, candidate term word] |
| 50 | community living.mp. [mp=title, abstract, heading word, drug trade name, original title, device manufacturer, drug manufacturer, device trade name, keyword heading word, floating subheading word, candidate term word] |
| 51 | community-living.mp. [mp=title, abstract, heading word, drug trade name, original title, device manufacturer, drug manufacturer, device trade name, keyword heading word, floating subheading word, candidate term word] |
| 52 | home-based.mp. [mp=title, abstract, heading word, drug trade name, original title, device manufacturer, drug manufacturer, device trade name, keyword heading word, floating subheading word, candidate term word] |
| 53 | home based.mp. [mp=title, abstract, heading word, drug trade name, original title, device manufacturer, drug manufacturer, device trade name, keyword heading word, floating subheading word, candidate term word] |
| 54 | (liv* adj6 house).mp. [mp=title, abstract, heading word, drug trade name, original title, device manufacturer, drug manufacturer, device trade name, keyword heading word, floating subheading word, candidate term word] |
| 55 | (resid* adj6 house).mp. [mp=title, abstract, heading word, drug trade name, original title, device manufacturer, drug manufacturer, device trade name, keyword heading word, floating subheading word, candidate term word] |
| 56 | (liv* adj6 home).mp. [mp=title, abstract, heading word, drug trade name, original title, device manufacturer, drug manufacturer, device trade name, keyword heading word, floating subheading word, candidate term word] |
| 57 | (resid* adj6 home).mp. [mp=title, abstract, heading word, drug trade name, original title, device manufacturer, drug manufacturer, device trade name, keyword heading word, floating subheading word, candidate term word] |
| 58 | (liv* adj6 community).mp. [mp=title, abstract, heading word, drug trade name, original title, device manufacturer, drug manufacturer, device trade name, keyword heading word, floating subheading word, candidate term word] |
| 59 | (resid* adj6 community).mp. [mp=title, abstract, heading word, drug trade name, original title, device manufacturer, drug manufacturer, device trade name, keyword heading word, floating subheading word, candidate term word] |
| 60 | house bound.mp. [mp=title, abstract, heading word, drug trade name, original title, device manufacturer, drug manufacturer, device trade name, keyword heading word, floating subheading word, candidate term word] |
| 61 | housebound.mp. [mp=title, abstract, heading word, drug trade name, original title, device manufacturer, drug manufacturer, device trade name, keyword heading word, floating subheading word, candidate term word] |
| 62 | homebound.mp. [mp=title, abstract, heading word, drug trade name, original title, device manufacturer, drug manufacturer, device trade name, keyword heading word, floating subheading word, candidate term word] |
| 63 | home bound.mp. [mp=title, abstract, heading word, drug trade name, original title, device manufacturer, drug manufacturer, device trade name, keyword heading word, floating subheading word, candidate term word] |
| 64 | exp Homebound Persons/ |
| 65 | 1 or 2 or 3 or 4 or 5 or 6 or 7 or 8 or 9 or 10 or 11 or 12 or 13 |
| 66 | 14 or 15 or 16 or 17 or 18 or 19 or 20 or 21 or 22 or 23 or 24 or 25 or 26 or 27 or 28 or 29 or 30 or 31 or 32 or 33 or 34 or 35 or 36 or 37 or 38 or 39 or 44 or 45 |
| 67 | 40 or 41 or 42 or 43 |
| 68 | 46 or 47 or 48 or 49 or 50 or 51 or 52 or 53 or 54 or 55 or 56 or 57 or 58 or 59 or 60 or 61 or 62 or 63 or 64 |
| 69 | 65 and 66 and 67 and 68 |
| 70 | limit 69 to (english language and yr="2009 - 2022") |

**Table 4: CINAHL**

| S67 | S62 AND S63 AND S64 AND S65 |
| --- | --- |
| S66 | S62 AND S63 AND S64 AND S65 |
| S65 | S44 OR S45 OR S46 OR S47 OR S48 OR S49 OR S50 OR S51 OR S52 OR S53 OR S54 OR S55 OR S56 OR S57 OR S58 OR S59 OR S60 OR S61 |
| S64 | S40 OR S41 OR S42 OR S43 |
| S63 | S14 OR S15 OR S16 OR S17 OR S18 OR S19 OR S20 OR S21 OR S22 OR S23 OR S24 OR S25 OR S26 OR S27 OR S28 OR S29 OR S30 OR S31 OR S32 OR S33 OR S34 OR S35 OR S36 OR S37 OR S38 OR S39 |
| S62 | S1 OR S2 OR S3 OR S4 OR S5 OR S6 OR S7 OR S8 OR S9 OR S10 OR S11 OR S12 OR S13 |
| S61 | (MH "Homebound Persons") |
| S60 | homebound |
| S59 | housebound |
| S58 | resid* N6 community |
| S57 | liv* N6 community |
| S56 | resid* N6 home |
| S55 | liv* N6 home |
| S54 | resid* N6 house |
| S53 | liv* N6 house |
| S52 | (MH "Home Rehabilitation+") |
| S51 | home-based |
| S50 | home based |
| S49 | community-living |
| S48 | community living |
| S47 | community-dwelling |
| S46 | community dwelling |
| S45 | (MH "Community Living+") |
| S44 | independent living |
| S43 | (MH "Alzheimer's Disease") |
| S42 | alzheimer* |
| S41 | (MH "Dementia+") OR (MH "Frontotemporal Dementia+") OR (MH "Dementia, Vascular+") OR (MH "Delirium, Dementia, Amnestic, Cognitive Disorders+") OR (MH "Dementia, Multi-Infarct") OR (MH "Lewy Body Disease") OR (MH "Dementia, Senile+") OR (MH "Dementia Patients") OR (MH "Dementia, Presenile+") |
| S40 | dement* |
| S39 | community care |
| S38 | gp |
| S37 | (MH "Primary Health Care") OR (MH "Physicians, Family") OR (MH "Multidisciplinary Care Team+") OR (MH "Health Services Needs and Demand+") OR (MH "Primary Nursing") OR (MH "Gerontologic Care") |
| S36 | primary care |
| S35 | (MH "Community Health Centers+") OR (MH "Community Health Nursing+") OR (MH "Community Health Workers") OR (MH "Community Health Services+") OR (MH "Health Services for Older Persons") |
| S34 | community health |
| S33 | (MH "Home Health Care+") OR (MH "Health Personnel+") OR (MH "Medical Care+") |
| S32 | primary healthcare |
| S31 | primary health care |
| S30 | (MH "Health Care Delivery+") OR (MH "Primary Health Care") |
| S29 | healthcare profession* |
| S28 | health care profession* |
| S27 | physical therap* |
| S26 | (MH "Physical Therapy Practice, Evidence-Based") OR (MH "Physical Therapy+") |
| S25 | physiotherap* |
| S24 | (MH "Occupational Therapy+") OR (MH "Occupational Therapy Assistants") OR (MH "Occupational Therapy Practice, Research-Based") OR (MH "Occupational Therapy Practice, Evidence-Based") OR (MH "Home Occupational Therapy") OR (MH "Occupational Therapy Service") |
| S23 | occupational therap* |
| S22 | (MH "Registered Nurses") OR (MH "Nurses by Role+") |
| S21 | (MH "State Nursing Organizations+") OR (MH "Nursing Organizations+") |
| S20 | nursing |
| S19 | nurse* |
| S18 | (MH "Geriatricians") |
| S17 | geriatrician* |
| S16 | physician* |
| S15 | (MH "Family Practice") OR (MH "Practical Nurses") OR (MH "Medical Practice+") |
| S14 | general practi* |
| S13 | nondrug |
| S12 | non-drug |
| S11 | (MH "Support, Psychosocial+") OR (MH "Psychosocial Intervention") |
| S10 | psychosocial |
| S9 | psycho-social |
| S8 | initiative* |
| S7 | innovation* |
| S6 | intervention* |
| S5 | (MH "Behavior Therapy+") |
| S4 | (MH "Internet-Based Intervention") OR (MH "Psychosocial Intervention") OR (MH "Early Intervention+") OR (MH "Intervention Trials") OR (MH "Nursing Interventions") |
| S3 | behavio* therap* |
| S2 | nonpharmacologic* |
| S1 | non-pharmacologic* |

**Table 5: COCHRANE CENTRAL**

| ID | Search |
| --- | --- |
| #1 | (dement*) OR (alzheimer*) |
| #2 | MeSH descriptor: [Dementia] explode all trees |
| #3 | MeSH descriptor: [Alzheimer Disease] explode all trees |
| #4 | #1 OR #2 OR #3 |
| #5 | (non-pharmacologic*) OR (nonpharmacologic*) OR ("behavio* therap*") OR (intervention*) OR (innovation*) |
| #6 | (initiative*) OR (psycho-social) OR (psychosocial) OR (nondrug) OR (non-drug) |
| #7 | MeSH descriptor: [Behavior Therapy] explode all trees |
| #8 | MeSH descriptor: [Psychosocial Intervention] explode all trees |
| #9 | #5 OR #6 OR #7 OR #8 |
| #10 | #4 AND #9 with Publication Year from 2009 to 2022, in Trials |
| #11 | ("general practi*") OR (physician*) OR (geriatrician*) OR (nurse*) OR (nursing) |
| #12 | ("occupational therap*") OR (physiotherap*) OR ("physical therap*") OR ("health care profession*") OR ("healthcare profession*") |
| #13 | ("primary health care") OR ("primary healthcare") OR ("community health") OR ("primary care") OR ("community care") |
| #14 | MeSH descriptor: [General Practice] explode all trees |
| #15 | MeSH descriptor: [Physicians] explode all trees |
| #16 | MeSH descriptor: [Geriatricians] explode all trees |
| #17 | MeSH descriptor: [Nursing] explode all trees |
| #18 | MeSH descriptor: [Occupational Therapy] explode all trees |
| #19 | MeSH descriptor: [Occupational Therapists] explode all trees |
| #20 | MeSH descriptor: [Physical Therapy Modalities] explode all trees |
| #21 | MeSH descriptor: [Physical Therapists] explode all trees |
| #22 | MeSH descriptor: [Primary Health Care] explode all trees |
| #23 | MeSH descriptor: [General Practitioners] explode all trees |
| #24 | MeSH descriptor: [Family Practice] explode all trees |
| #25 | MeSH descriptor: [Community Health Centers] explode all trees |
| #26 | MeSH descriptor: [Community Health Nursing] explode all trees |
| #27 | MeSH descriptor: [Geriatric Nursing] explode all trees |
| #28 | #11 OR #12 OR #13 OR #14 OR #15 OR #16 OR #17 OR #18 OR #19 OR #20 OR #21 OR #22 OR #23 OR #24 OR #25 OR #26 OR #27 |
| #29 | #4 AND #9 AND #28 with Publication Year from 2009 to 2022, in Trials |
| #30 | home* |
| #31 | communit* |
| #32 | house* |
| #33 | #30 OR #31 OR #32 |
| #34 | #4 AND #9 AND #28 AND #33 with Publication Year from 2009 to 2022, in Trials |
| #35 | MeSH descriptor: [Independent Living] explode all trees |
| #36 | MeSH descriptor: [Homebound Persons] explode all trees |
| #37 | #30 OR #31 OR #32 OR #35 OR #36 |
| #38 | #4 AND #9 AND #28 AND #37 with Publication Year from 2009 to 2022, in Trials |
| #39 | MeSH descriptor: [Internet-Based Intervention] explode all trees |
| #40 | MeSH descriptor: [Clinical Trial] explode all trees |
| #41 | MeSH descriptor: [Health Education] explode all trees |
| #42 | #9 OR #39 OR #40 OR #41 |
| #43 | #4 AND #42 AND #28 AND #37 with Publication Year from 2009 to 2022, in Trials |
