# Supplementary material for: Interventions delivered by primary or community healthcare professionals to support people living at home with dementia with activities of daily living: a systematic review and narrative synthesis
Source: BMC Geriatr. 2024 Oct 23;24:860. doi: 10.1186/s12877-024-05465-5 (PMC11515696; doi:10.1186/s12877-024-05465-5)
Supplement: Supplementary file 2 — Supplementary Material 2 [file 12877_2024_5465_MOESM2_ESM.docx]

**DEMCON STUDY – DATA SCREENING AND EXTRACTION FORM**

**Part 1: Citation, title, and abstract screening**

| **Reviewer 1** | | | |
| --- | --- | --- | --- |
|  | **Yes** | **No** | **Unsure** |
| Does the **citation/title/abstract** meet all the inclusion criteria? |  |  |  |
| If yes or unsure, proceed to full-text screening (part 2).  If no, please note the reason for its exclusion below. | | | |
|  | | | |
| **Reviewer 2** | | | |
|  | **Yes** | **No** | **Unsure** |
| Does the **citation/title/abstract** meet all the inclusion criteria? |  |  |  |
| If yes or unsure, proceed to full-text screening (part 2).  If no, please note the reason for its exclusion below. | | | |
|  | | | |
| **Reviewer 3 (if reviewers 1 and 2 disagree and cannot reach agreement)** | | | |
|  | **Yes** | **No** | **Unsure** |
| Does the **citation/title/abstract** meet all the inclusion criteria? |  |  |  |
| If yes or unsure, proceed to full-text screening (part 2).  If no, please note the reason for its exclusion below. | | | |
|  | | | |

**Part 2: Full-text screening**

| **Reviewer 1** | | | |
| --- | --- | --- | --- |
|  | **Yes** | **No** | **Unsure** |
| Does the **full text** meet all the inclusion criteria? |  |  |  |
| If yes proceed to data extraction (part 3).  If no, please note the reason for its exclusion below. | | | |
|  | | | |
| **Reviewer 2** | | | |
|  | **Yes** | **No** | **Unsure** |
| Does the **full text** meet all the inclusion criteria? |  |  |  |
| If yes proceed to data extraction (part 3).  If no, please note the reason for its exclusion below. | | | |
|  | | | |
| **Reviewer 3 (if reviewers 1 and 2 disagree or are unsure and cannot reach agreement)** | | | |
|  | **Yes** | **No** | **Unsure** |
| Does the **full text** meet all the inclusion criteria? |  |  |  |
| If yes proceed to data extraction (part 3).  If no, please note the reason for its exclusion below. | | | |
|  | | | |

**Part 3: Data Extraction**

| Reviewed by (initials) |  |
| --- | --- |
| Date reviewed (dd/mm/yy) |  |

1. **Article details**

| Author(s) |  |
| --- | --- |
| Year of publication |  |
| Title |  |
| Journal |  |
| Volume |  |
| Issue |  |
| Pages |  |
| DOI |  |

1. **Study design/details**

| **Item** | **Details** |
| --- | --- |
| 1. Aim of the paper |  |
| 1. Focus (in terms of intervention development/delivery/evaluation) |  |
| 1. Design/study type |  |
| 1. Location (geographical) |  |
| 1. Setting |  |
| 1. Study period (dates of data collection) |  |
| 1. Participants/care recipient group |  |
| 1. Type and number of study locations (e.g. health and social care organisations, teams etc.) |  |
| 1. Sample size (individuals, health and social care organisations, teams etc.) |  |
| 1. IADL/ADL focus^1^ |  |
| 1. Key author conclusions (from abstract) |  |

^1^IADL (Instrumental Activities of Daily Living) and Activities of Daily Living (ADL).

1. **Brief description of the intervention**

| **Item** | **Details** |
| --- | --- |
| 1. Brief name/phrase describing intervention |  |
| 1. Details of intervention and comparator groups |  |
| 1. Key components of the intervention |  |
| 1. Who provided intervention (background, expertise, and training) |  |
| 1. Mode of delivery (e.g. face-to-face, telephone, online) |  |
| 1. Intervention intensity (number, duration, and frequency of contact sessions, if applicable) |  |

1. **Evidence to inform guiding principles/intervention planning**

The following domains have been identified based on a review of what data has previously been extracted in other studies that have used a person-based approach. Please enter all relevant information relating to the development, delivery, and evaluation of interventions. This information may be explicit or implicit in the publications. Relevant information that does not fit in domains 1-8 should be entered in row ‘9.’

|  | **Domain** | **Extracted information (verbatim)** |
| --- | --- | --- |
| 1 | Relevant contextual or environmental factors |  |
| 2 | Target behaviours (*behaviour that has been chosen or targeted for change*) |  |
| 3 | Barrier to target behaviour (*include details of methods to address barriers*) |  |
| 4 | Facilitator to target behaviour (*include ways in which these could be incorporated*) |  |
| 5 | Important influences on target behaviours |  |
| 6 | Barriers of the intervention^1^ |  |
| 7 | Facilitators of the intervention^1^ |  |
| 8 | Promising intervention features/ingredients (*which may be designed to promote/incorporate facilitators and overcome/address barriers*) |  |
| 9 | Other relevant findings reported in the paper |  |

**^1^**Barriers/facilitators affecting uptake, acceptability, adherence, feasibility, positive behaviour change, efficacy, and engagement (how these were or could be addressed/used).

1. **Please make a note of any initial potential themes identified during data extraction**

|  |
| --- |
